# Origami with small molecules: exploiting the C–F bond as a conformational tool

**DOI:** 10.3762/bjoc.21.54

**Published:** 2025-04-02

**Authors:** Patrick Ryan, Ramsha Iftikhar, Luke Hunter

**Affiliations:** 1 School of Chemistry, The University of New South Wales (UNSW), Sydney 2052, Australia

**Keywords:** conformational analysis, medicinal chemistry, organofluorine chemistry, stereoselective fluorination

## Abstract

When present within an organic molecule, the C–F bond tends to align in predictable ways with neighbouring functional groups, due to stereoelectronic effects such as hyperconjugation and electrostatic attraction/repulsion. These fluorine-derived conformational effects have been exploited to control the shapes, and thereby enhance the properties, of a wide variety of functional molecules including pharmaceutical agents, liquid crystals, fragrance chemicals, organocatalysts, and peptides. This comprehensive review summarises developments in this field during the period 2010–2024.

## Introduction

In the art of origami, a practitioner takes a piece of paper and imposes a series of folds in order to transform it into an object that has an intricate three-dimensional shape. This concept can also be applied in the molecular world. Starting with a flexible small molecule, a practitioner can impose certain changes to its chemical composition such that the new molecule has a better-defined three-dimensional shape. Such “small molecule origami” can offer practical benefits. For example, if a drug molecule is pre-organised into the target-binding conformation, it should exhibit the desirable twin characteristics of high potency (since target binding will incur little entropic cost) and high selectivity (since off-target interactions will be minimised) [1]. There are several methods by which the conformations of small molecules can be controlled, but in this review we will focus upon one particular method, which is the installation of fluorine atoms into the structure.

**Figure 1:**
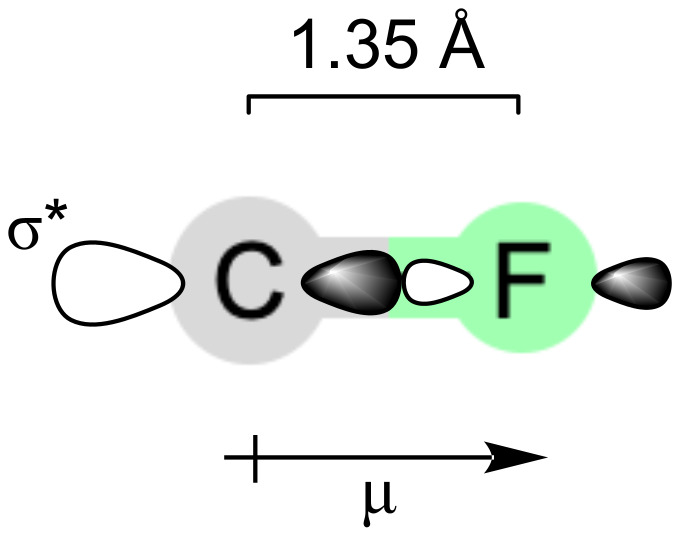
Fundamental characteristics of the C–F bond.

The C–F bond has certain fundamental characteristics that enable it to serve as an effective conformational tool (Figure 1) [2–4]. First, the C–F bond is quite short at only ≈1.35 Å (cf. ≈1.09 Å for C–H, or ≈1.43 Å for C–O). The short length of the C–F bond, and the compact size of the fluorine atom itself, means that fluorine can be incorporated into an organic molecule as a replacement for hydrogen without drastically altering the molecular volume. Second, the C–F bond is highly polarised. This means that any molecular conformation in which the C–F dipole is oriented antiparallel to another dipole within the molecule, or in which the fluorine atom is located close to a positively charged atom, will be stabilised. Third, the C–F bond has a low-lying σ* antibonding orbital, the larger lobe of which is located behind the carbon atom. This empty orbital is available to mix with any nearby filled orbital in a process known as hyperconjugation, and any molecular conformation in which such mixing can occur, will be stabilised [5–7].

Putting all these concepts together, it is possible to conceive of a process whereby a conformationally flexible lead compound is rigidified in a predictable and desired way, by decorating it with an appropriate pattern of fluorine substituents.

The purpose of this review is to examine cases where this idea has been put into practice. This topic has previously been reviewed [8], but in the time that has elapsed since that prior publication the field has expanded considerably and so we believe that an updated account is warranted. In the present review, we have chosen to organise the material according to the functional group near to which the fluorine substituent can be introduced. We will start with the simplest scaffolds – alkanes – then we will progress to ethers, alcohols, sugars, amines (and their derivatives), carbonyl compounds, peptides, and finally sulfur-containing compounds. By arranging the material in this way, we hope that newcomers to the field might be able to readily envisage ways to apply these concepts to their own scaffolds of interest.

## Review

### 1 Alkanes

The simplest organic scaffolds are the alkanes. In such molecules, C–C bond rotations often have low energy barriers, and they often deliver conformers that are similar in energy, and this means that many alkanes have considerable conformational flexibility. In this section, we will investigate the conformational outcomes that follow from replacing one or more hydrogens of an alkane with fluorine. Depending upon the precise fluorination pattern, different conformational outcomes will follow; usually, but not always, greater rigidity is seen. This section will commence by examining linear alkanes, then it will move on to examine cycloalkanes.

**Figure 2:**
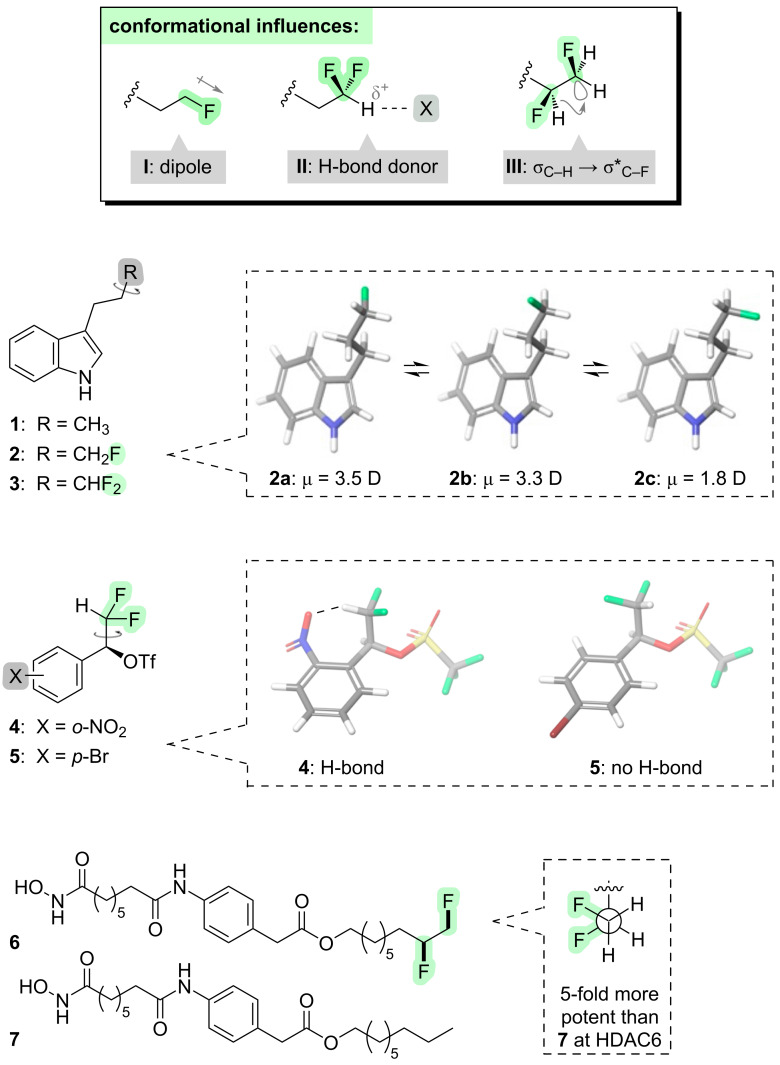
Incorporation of fluorine at the end of an alkyl chain.

In the case of linear alkanes, we will first consider what happens if fluorine is introduced at the end of the chain. The installation of fluorine converts the alkyl chain from a non-polar into a polar motif (**I**, Figure 2). This has several implications. For example, if the C–C(F) bond rotates, the orientation of the terminal C–F bond dipole changes, and this can alter the overall dipole moment of the molecule. Indoles **1**–**3** illustrate this point (Figure 2) [9]. The non-fluorinated indole **1** has an unvarying molecular dipole moment of 1.90 D. In contrast, the monofluorinated analogue **2** can access three different staggered rotamers **2a**–**c** about the C–C(F) bond; all three of these rotamers have similar energies, but their molecular dipole moments vary considerably depending on whether the C–F dipole is aligned with or against the dipole of the indole moiety. A similar phenomenon occurs with the difluorinated analogue **3**. The fluorinated molecules **2** and **3** can be said to have a “chameleonic” character [9–14]: they have the ability to change polarity to suit their environment. This is a potentially valuable property in the context of drug design, because chameleonic fluorinated molecules such as compounds **2** and **3** might be expected to pass more easily through cell membranes, a manoeuvre which requires some level of solubility in both aqueous and organic environments.

Another consequence of introducing polar C–F bonds at the end of an alkyl chain, is that the terminal C–H bond also becomes polarised. In the case of the difluoromethyl group, the terminal hydrogen bears a partial positive charge and is able to act as a H-bond donor (**II**, Figure 2). This can influence the conformation of the molecule if there is a H-bond acceptor suitably positioned elsewhere in the molecule (e.g., **4** vs **5**, Figure 2) [15–17]. The ability of the difluoromethyl group to serve as a H-bond donor has also proven to be useful for optimising drug–target interactions [18–20], but since that application does not involve conformational control it is outside the scope of this review.

Another way to fluorinate the end of an alkyl chain is with a 1,2-difluoro pattern. The vicinal difluoro motif is able to sample different rotamers (e.g., with the C–F bonds aligned either *gauche* or *anti*). Crucially however, such rotamers have different energies. When the C–F bonds are aligned *gauche*, the vacant σ* orbital of each C–F bond is able to mix with the filled σ orbital of an adjacent C–H bond, and this hyperconjugative interaction stabilises the *gauche* conformer (**III**, Figure 2). The *anti* conformer does not benefit from this hyperconjugative stabilisation, and it is ≈1 kcal·mol^−1^ higher in energy. Thus, we now have a situation where the fluorinated structural motif is not a passive chameleon as was seen above for the 1,1-difluoro pattern, but rather it exerts its own conformational character [21]. The 1,2-difluoro motif has been exploited in the design of bioactive molecules [22–23] such as the histone deacetylase (HDAC) inhibitors **6** and **7** (Figure 2). The presence of the 1,2-difluoro moiety in **6** leads to greater potency and selectivity for certain HDAC isoforms, attributable to the higher polarity of the fluorinated motif with its *gauche*-aligned C–F bonds [22].

**Figure 3:**
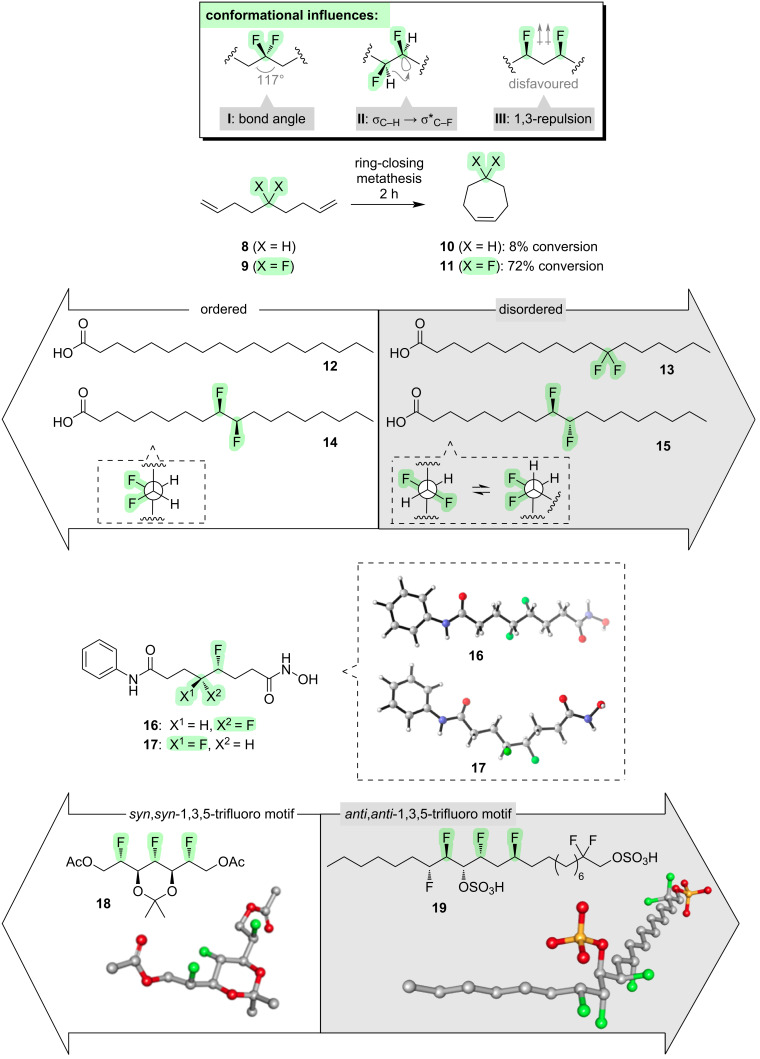
Incorporation of fluorine into the middle of a linear alkyl chain.

We will now consider what happens when fluorine is introduced into the middle of an alkyl chain. If two fluorines are attached to the same carbon (i.e., a 1,1-difluoro pattern), a subtle but important perturbation occurs to the shape of the alkyl chain: the C–C(F_2_)–C angle widens to ≈117° (**I**, Figure 3). This can be attributed to the electron-withdrawing character of the fluorine atoms, which accumulates high electron density within a small volume and allows the C–C bonds to spread further apart from one another [2]. An alternative explanation for this phenomenon is provided by Bent’s rule [24], which predicts that the C–F bonds will have greater p character and the C(F_2_)–C bonds will have greater s character, with an accompanying deviation of the central carbon atom away from a perfectly tetrahedral shape. A functional outcome of C–C(F_2_)–C angle widening in *gem*-difluoroalkanes is seen in the rates of the ring-closing methathesis reactions of the dienes **8** and **9** (Figure 3) [25]. The fluorinated substrate **9** cyclises much more efficiently than the non-fluorinated substrate **8**, and this was attributed to a thermodynamic effect, i.e., a more favourable accommodation of a wider C–C(F_2_)–C angle within the cyclic product **11**. Another illustration of the geometric perturbation caused by the wider C–C(F_2_)–C angle comes from the stearic acids **12** and **13** (Figure 3); the *gem*-difluorinated analogue **13** has substantially greater conformational disorder compared to the non-fluorinated stearic acid **12** (Figure 3) [26].

If two fluorines are introduced into the middle of an alkyl chain in a 1,2-pattern, several competing factors arise that can influence the molecular conformation [27]. One factor is hyperconjugation: conformations in which the σ*_C–F_ orbitals are aligned with either σ_C–H_ orbitals (**II**, Figure 3), or, to a lesser extent, σ_C–C_ orbitals, will be favoured. Another factor is simple sterics: conformations in which the flanking alkyl moieties are further apart from one another will be favoured. A third factor is polarity: for example, conformations in which the two C–F bonds are aligned *gauche* will be favoured in water due to their high molecular dipole moment. A final layer of complexity is afforded by the stereochemistry of the 1,2-difluoroalkane motif: the various conformational factors described above will aggregate differently depending upon whether the 1,2-difluoro stereochemistry is *threo* or *erythro*. For example, the diastereoisomeric difluorinated stearic acids **14** and **15** (Figure 3) are found to have very different physical properties [28]. When deposited into a monolayer above a water phase, the *threo*-isomer **14** occupies a small molecular area and packs efficiently, and this was attributed to the ready ability of this stereoisomer to adopt an extended alkyl chain in which the C–F bonds are aligned *gauche*. In contrast, the *erythro*-isomer **15** occupies a larger molecular area and requires a higher pressure to achieve a monoloayer, and this was attributed to the partial tendency of this stereoisomer to adopt a bent alkyl chain. Another example of the use of the 1,2-difluoro moiety to influence the shape of an alkyl chain is seen in the HDAC inhibitors **16** and **17** (Figure 3) [29]. The *threo*-isomer **16** is found to be consistently more potent across a panel of HDAC isoforms than the *erythro*-isomer **17**, and this was taken as evidence that an extended zigzag conformation of the alkyl chain is required for binding to HDAC.

If two fluorines are introduced into the middle of an alkyl chain in a 1,3-pattern, a new conformational effect emerges. The 1,3-C–F bonds tend to avoid a parallel alignment, due to dipolar repulsion (**III**, Figure 3) [30–32]. This phenomenon can be harnessed to control molecular conformations in a predictable way, and once again the stereochemistry is important. For example, compare the natural product analogues **18** and **19** (Figure 3) [30,33]. Compound **18** contains a 1,3,5-trifluoroalkane moiety with *syn*,*syn*-stereochemistry, and it is found to adopt a bent alkyl chain which is necessary for the avoidance of parallel 1,3-difluoro alignments [30]. Compound **19** also contains a 1,3,5-trifluoroalkane moiety, but it has *anti*,*anti*-stereochemistry and now this portion of the alkyl chain is able to adopt an extended zigzag conformation without incurring any parallel 1,3-difluoro alignments [33].

Other fluorine patterns have been shown to control molecular conformation when embedded in the middle of an alkyl chain [34], for example the 1,1,3-trifluoro motif [35], the 1,1,3,3,-tetrafluoro motif [36] and the 1,1,4,4-tetrafluoro motif [36]. In general, the conformational outcomes in such systems can be understood in terms of an aggregate of the various conformational influences already described (i.e., **I**–**III**, Figure 3).

**Figure 4:**
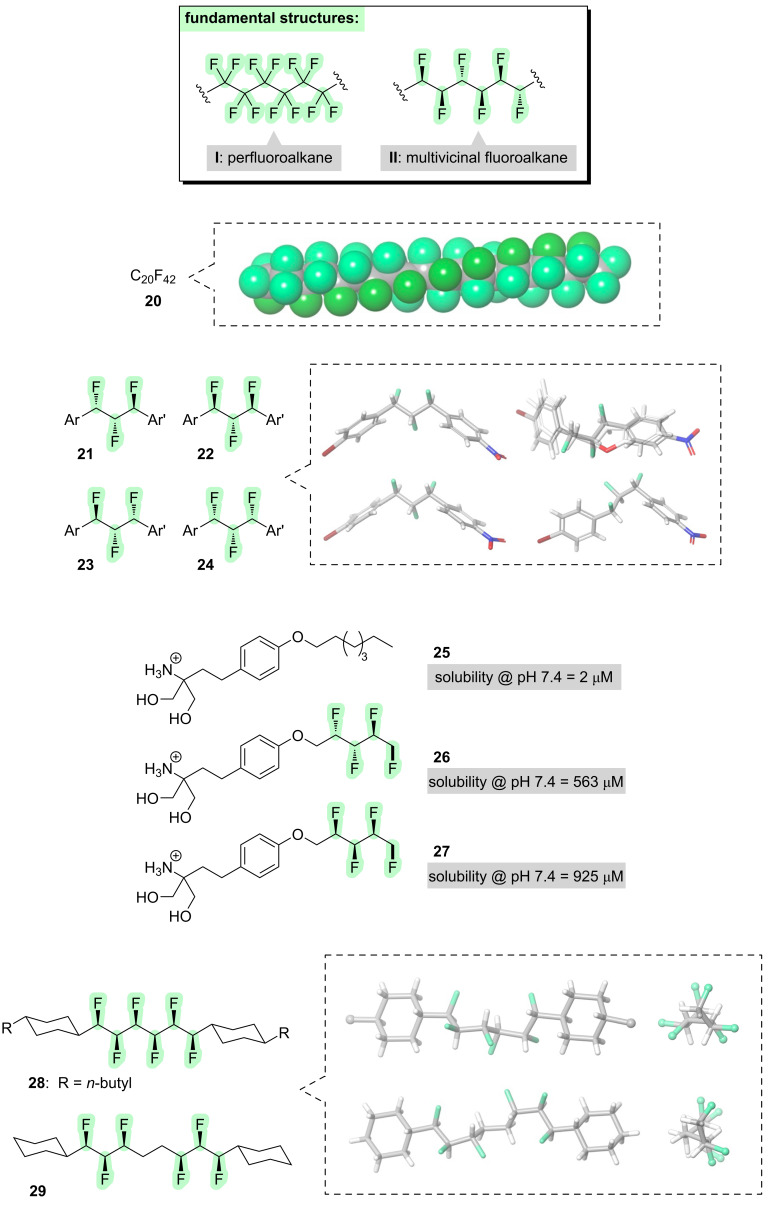
Incorporation of fluorine across much, or all, of a linear alkyl chain.

We will now consider what happens when fluorine is introduced across much*,* or all, of an alkyl chain. The extreme case is a perfluoroalkane, in which every hydrogen is replaced with a fluorine (e.g., **I**, Figure 4). Perfluoroalkanes are not usually thought of in terms of controllable molecular conformations, but there is one aspect of their conformational behaviour that merits discussion here. The carbon chain in perfluoroalkanes deviates in a precise way from an ideal zigzag conformation: each C–C–C–C dihedral angle is ≈165°, and when propagated along the chain this slight deviation from antiperiplanarity leads to a gradual helical twist (**20**, Figure 4) [37]. The enantiomeric helix is also possible. Various explanations have been offered for this helical propensity, but the current understanding is that the slight twist enables better σ_C–C_ → σ*_C–F_ hyperconjugation [38].

If just one fluorine atom is attached to every carbon of an alkyl chain (e.g., **II**, Figure 4), then a structure results that is conceptually intermediate between alkanes and perfluoroalkanes. Such structures, dubbed “multivicinal fluoroalkanes”, have interesting conformational properties that are dependent upon the stereochemistry [39–42]. For example, the diastereoisomeric 1,2,3-trifluoroalkanes **21**–**24** (Figure 4) adopt distinct conformations, governed in each case by the avoidance of parallel 1,3-C–F bonds and the maximisation of σ_C–H_ → σ*_C–F_ hyperconjugation [43]. Notably, the overall shape of diastereoisomer **22** closely mimics that of 2-benzyl-2,3-dihydrobenzofuran (overlaid in Figure 4), a structural motif that is commonly found within bioactive natural products.

Having seen that the conformations of multivicinal fluoroalkanes can be altered by changing the stereochemistry, it follows that other physical properties can be altered, too [44]. For example, consider compounds **26** and **27** (Figure 4), which are fluorinated analogues of the multiple sclerosis drug gilenya (**25**) [45]. The *syn*,*anti*-fluorinated stereoisomer **26** has a much higher water solubility than gilenya (**25**), attributable to the presence of new polar bonds in **26** (although the different alkyl chain lengths in **25** and **26** should also be acknowledged). Notably, the all-*syn* fluorinated stereoisomer **27** displays a further increase in water solubility, even compared to the *syn*,*anti*-stereoisomer **26**, highlighting the dramatic impact that a seemingly minor stereochemical change can have upon physical properties.

Another functional context in which multivicinal fluoroalkanes have been explored is in liquid crystals [8,40]. Liquid crystalline materials require the molecules to be rod-shaped, and to have a dipole moment that is oriented perpendicular to the long axis of the molecule. The strategic incorporation of multi-fluorine patterns has been investigated as a method for simultaneously modulating both of these molecular characteristics, i.e., the molecular shape and the dipole moment. For example, compound **28** (Figure 4) contains six vicinal fluorines with all-*syn* stereochemistry [46]. This molecule adopts a helical conformation which maximises 1,2-difluoro *gauche* alignments and minimises 1,3-difluoro parallel alignments. (Note that the helix in this case is much “tighter” than the perfluoroalkane helix described above.) The helical conformation of **28** leads to a low molecular dipole moment, because every C–F bond can be paired with another that has the opposite orientation. In contrast, compound **29** (Figure 4) divides the six fluorines into two groups of three, separated by an ethylene spacer [47]. The same helical conformation is maintained, but the interruption of the fluorine pattern means that the C–F dipoles no longer all cancel each other out, and this results in a very high calculated molecular dipole (μ = 7.15 D).

Having concluded our survey of linear alkanes, we will now focus on cyclic systems [48].

**Figure 5:**
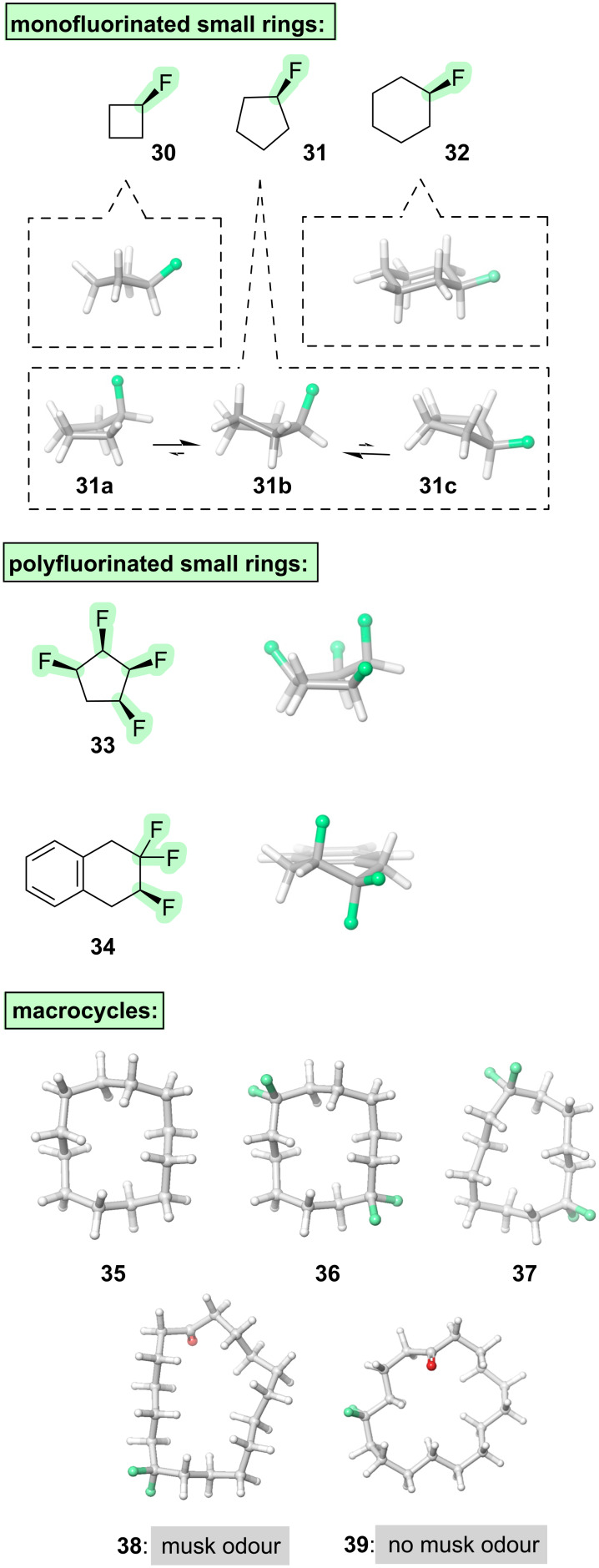
Incorporation of fluorine into cycloalkanes.

When fluorine is introduced into a small cycloalkane (Figure 5), the preferred pucker is determined through a competition between hyperconjugation (e.g., σ_C–H_ → σ*_C–F_) and steric effects. For fluorocyclobutane (**30**) and fluorocyclohexane (**32**), the preferred conformation is the one in which fluorine is equatorial [48], with sterics playing the dominant role. In fluorocyclopentane (**31**), the preferred conformation has historically been difficult to conclusively pin down, with different studies identifying different candidates for the global minimum including an envelope with fluorine axial (**31a**); an envelope with fluorine equatorial (**31c**); or, most recently, an intermediate twist conformation (**31b**) [48]. Multiple fluorines can sometimes provide better control. For example, all-*syn*-1,2,3,4-tetrafluorocyclopentane (**33**) appears to prefer an envelope conformation with fluorine in the axial position [49]. Another example of conformational control via multiple fluorines is seen with the trifluorinated cyclohexene **34**: this molecule prefers the half-chair conformation in which σ_C–H_ → σ*_C–F_ hyperconjugation with the flanking methylene groups is maximised (Figure 5) [50].

When discussing small-ring cycloalkanes, it is impossible to ignore a substantial research effort that has focused upon multifluorinated cyclohexanes [40,48,51]. Notable examples that have been synthesised and studied include all-*syn* 1,3,4-trifluorohexane [52], 1,1,3,3-tetrafluorocyclohexane [53], all-*syn*-1,2,3,4-tetrafluorocyclohexane [54], all-*syn*-1,2,4,5-tetrafluorocyclohexane [55–61], and various stereoisomers of 1,2,3,4,5,6-hexafluorocyclohexane [62] including the iconic all-*syn*-isomer [63–71]. These molecules have been shown to have a variety of fascinating properties and applications, mostly associated with their very high molecular dipoles. However, since the fluorination patterns in these cases do not really control or alter the ring pucker, they are outside the scope of this review.

Finally, we will examine larger-ring cycloalkanes. Large rings have more degrees of freedom than small rings, and hence they offer interesting opportunities and challenges in terms of conformational control. For example, consider cyclododecane (**35**, Figure 5). This molecule has a global minimum energy conformation that features a square shape, but this conformation suffers from steric clashes between 1,4-pairs of hydrogen atoms on the inside of the macrocycle, and so it is only slightly preferred over multiple other possible conformations. Fluorine has the ability to stabilise the square conformation. The key consideration is that 1,1-difluoroalkanes have a wide C–C(F_2_)–C angle, as previously discussed; this wide angle is favourably accommodated at the corner positions of the square shape (e.g., **36**, Figure 5) because it alleviates the transannular hydrogen clashes [72]. This effect proves to be quite general: it can be harnessed to force the cyclododecane macrocycle into different shapes by positioning two CF_2_ groups in different relative locations around the macrocycle (e.g., **37**, Figure 5), and it can be applied to larger macrocycles too [73]. Most strikingly, this effect has been exploited to modulate the aromas of fragrance compounds (e.g., compounds **38** and **39**, Figure 5) by fine-tuning the shape of the macrocycle [74–75].

### 2 Ethers

We now turn our attention from alkanes to what is arguably the simplest heteroatom-containing functional group: the ether. The presence of oxygen within a carbon chain offers several new opportunities for engagement by an introduced fluorine atom.

**Figure 6:**
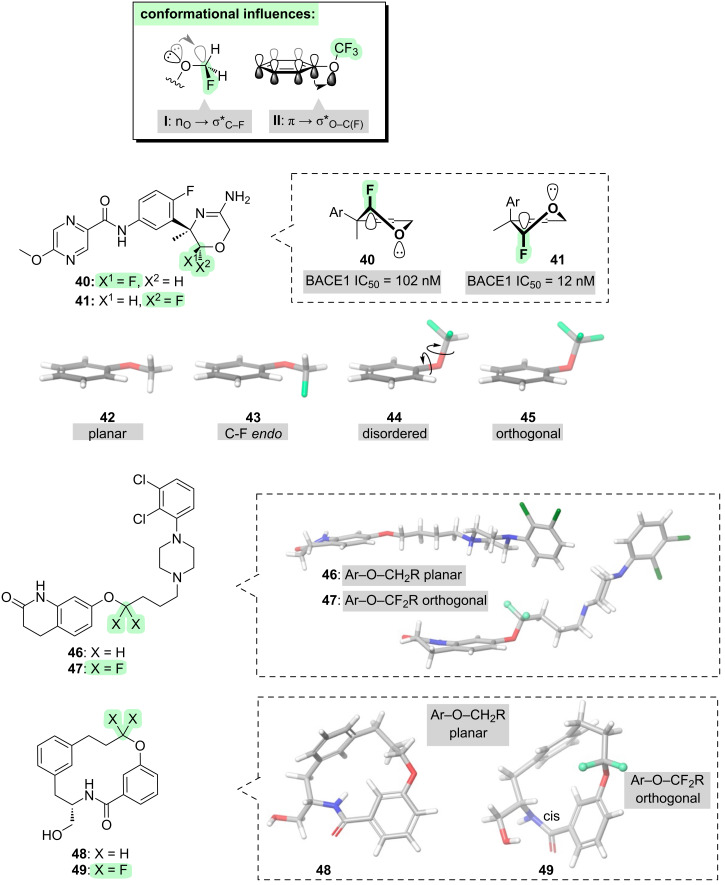
Conformational effects of introducing fluorine into an ether (geminal to oxygen).

First, we will consider what happens if fluorine is introduced onto one of the carbons that is directly attached to oxygen (Figure 6). This makes possible a hyperconjugative interaction between a lone pair on oxygen and the σ* orbital of the C–F bond (**I**, Figure 6) [76]. This interaction biases the rotational profile about the O–C(F) bond, such that the *endo* orientation of the C–F bond (as depicted in **I**, Figure 6) is lower in energy than the *exo* conformer (not shown). This is an example of the anomeric effect [77].

The anomeric effect can be visualised perhaps most clearly when the ether moiety is embedded within a small ring [78–79]. For example, consider the diastereoisomeric BACE-1 inhibitors **40** and **41** (Figure 6). The preferred ring pucker for each of **40**, **41** positions the C–F bond in an axial position, allowing n_O_ → σ*_C–F_ hyperconjugation. The shape adopted by diastereoisomer **41**, with a pseudoaxial orientation of the pendant aryl moiety, is better for target-binding and hence **41** is a ≈10-fold more potent inhibitor of BACE-1 than **40**.

Several further examples of cyclic ethers will be examined in section 5 (sugars).

The anomeric effect applies in acyclic ethers, too. Consider the non-fluorinated scaffold, Ph–O–CH_3_ (**42**, Figure 6). This molecule preferentially adopts a planar conformation [80]. The planar conformation suffers slightly from a steric clash between the methyl group and an aryl hydrogen, but this is outweighed by the favourable conjugation of the π-system with the lone pair in the 2p orbital of the sp^2^-hybridised oxygen atom. When one fluorine is introduced into this scaffold in the form of a fluoromethyl group (**43**, Figure 6), the gross conformation is little changed, but notably the fluorine preferentially resides at an *endo* position (oriented back towards the aryl moiety) which enables n_O_ → σ*_C–F_ hyperconjugation [81].

Progressing to the difluoromethyl system (**44**, Figure 6): now there is a very different situation because the molecule is quite disordered [81–82]. Rotamers about both Ar–O and (Ar)O–C bonds are now observed with similar energies; this notably includes conformations in which the difluoromethyl group is orthogonal to the aryl plane (as depicted in Figure 6). The accessibility of the orthogonoal conformation of **44** can be rationalised by a new effect, namely, hyperconjugation between the π-system and the σ* orbital of the O–C(F) bond (**II**, Figure 6). Overall, **44** is a disordered molecule which has potentially useful chameleonic polarity (and also lipophilic H-bond-donor ability).

Progressing to the trifluoromethyl system (**45**, Figure 6): now there is an even stronger tendency for the fluorinated group to be oriented orthogonal to the aryl plane [80]. This can be explained by two factors. First, π → σ*_O–C(F)_ hyperconjugation is stronger in the trifluoromethyl case. Second, the steric demand of the trifluoromethyl group would cause a significant clash with the aryl moiety in the planar conformation.

The increased accessibility of the orthogonal conformation of highly fluorinated ethers is also seen when Ar–O–CF_2_- is a linker moiety. For example, consider compound **47** (Figure 6), which is a fluorinated analogue of the antipsychotic drug, aripiprazole (**46**). The presence of the fluorine atoms in **47** causes a change in the conformation of the aryl ether moiety from planar in **46** [83] to orthogonal in **47** [84], leading to different overall molecular shapes for compounds **46** and **47**. Another illustration of this phenomenon is seen with the macrocycles **48** and **49**, which are simplified analogues of a known BACE-1 inhibitor [85]. In the non-fluorinated macrocycle **48**, the aryl ether moiety features a planar conformation, whereas in the fluorinated macrocycle **49**, the aryl ether moiety adopts an orthogonal conformation which necessitates substantial reorganisation elsewhere in the macrocycle including a *cis*-amide on the opposite side of the molecule.

**Figure 7:**
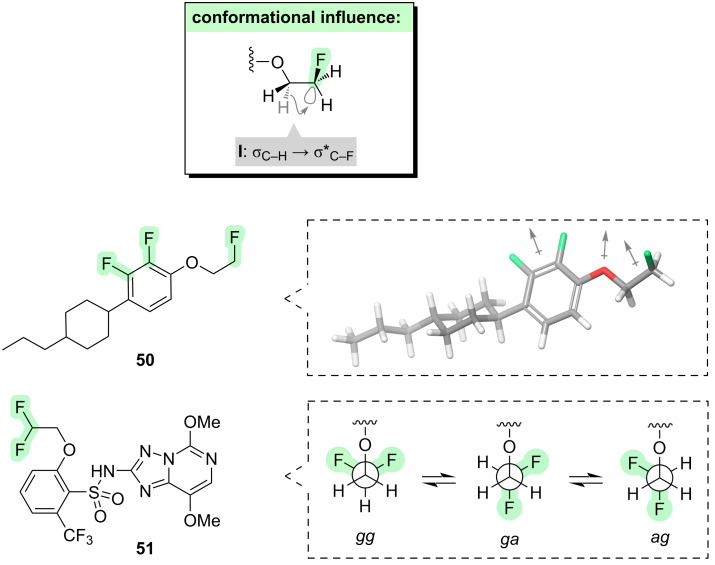
Conformational effects of introducing fluorine into an ether (vicinal to oxygen).

We now consider what happens if fluorine is introduced onto a carbon that is one atom further away from the ether oxygen (Figure 7). So doing introduces the opportunity for a new stereoelectronic phenomenon: a *gauche* effect for the O–C–C–F moiety (**I**, Figure 7). If the vicinal C–O and C–F bonds align *gauche* to one another, two stabilising hyperconjugative interactions can take place (i.e*.,* σ_C–H_ → σ*_C–F_ and σ_C–H_ → σ*_C–O_, with the former likely to be stronger). This is only a subtle effect, with the *gauche* conformer just ≈0.3 kcal·mol^−1^ lower in energy than the *anti* conformer (not shown) [86], but it can nevertheless have a significant impact upon molecular properties.

The fluorine–oxygen *gauche* effect has been exploited to influence the properties of liquid crystals [86–87]. For example, the vicinal fluoroether **50** (Figure 7) can adopt a low-energy conformation in which the terminal C–F bond aligns *gauche* to the vicinal C–O bond. This orientation of the C–F bond enlarges the overall molecular dipole moment which is oriented perpendicular to the long axis of the molecule; as described earlier, this an important feature in liquid crystalline materials.

Now consider the case where two fluorine atoms are introduced onto the same carbon (e.g., **51**, Figure 7) [88–89]. This situation is more complex. The *gg* conformer, in which both fluorines are *gauche* to oxygen, benefits from multiple hyperconjugative interactions (i.e., σ_C–H_ → σ*_C–F_ and σ_C–H_ → σ*_C–O_) and it is also the most polar conformation, so it is favoured in water. However, the *gg* conformer suffers from Lewis F···O repulsion. In contrast, the *ga* and *ag* conformers have less hyperconjugation but also less F···O repulsion, and are less polar. Since all three conformers are close in energy, the difluoroethyl ether moiety in **51** can be considered to have chameleonic polarity.

### 3 Alcohols

We now progress from ethers (section 2) to a closely related class of molecules, namely, the alcohols. The focus here in section 3 will be mostly on simple examples; more complex poly-ol examples will be discussed in section 4 (sugars).

**Figure 8:**
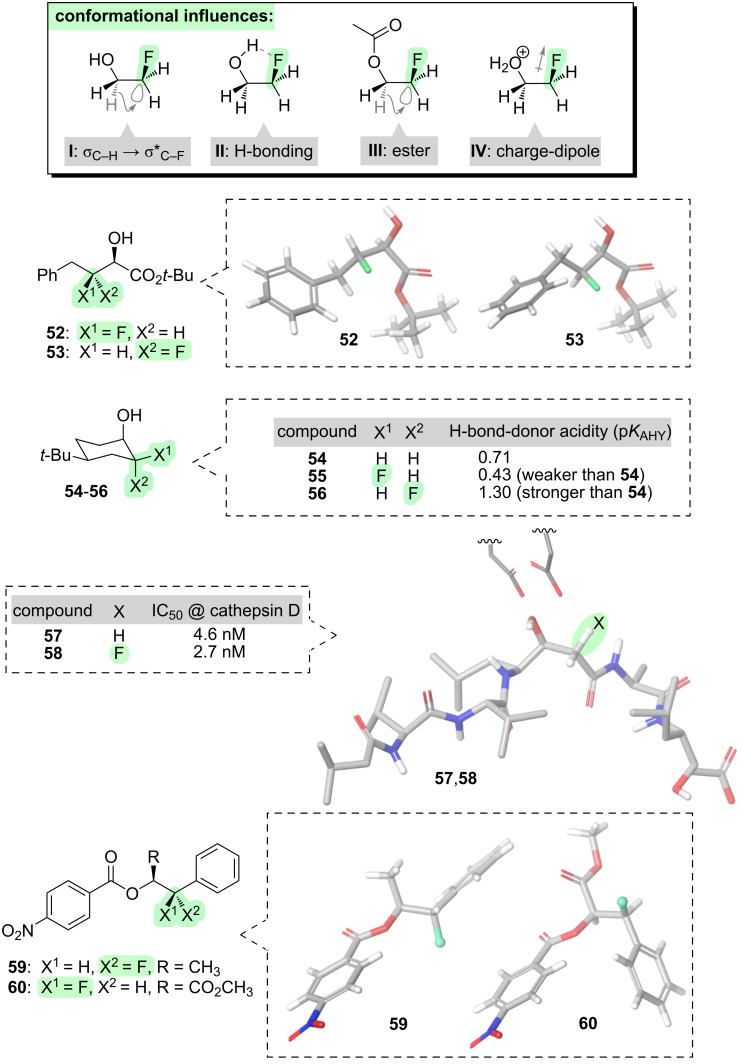
Effects of introducing fluorine into alcohols (and their derivatives).

Taking the example of a simple alcohol such as ethanol, consider what happens when a fluorine atom is introduced vicinal to the hydroxy group (Figure 8). As was seen with ethers (**I**, Figure 7), there is again a subtle preference for the O–C–C–F motif to adopt a *gauche* conformation [44]. This is attributable in part to the hyperconjugation phenomenon (**I**, Figure 8). In several published crystal structures of vicinal fluorohydrins, the F–C–C–O motif adopts a *gauche* conformation as expected [90–93]. This *gauche* preference can induce different overall shapes to be adopted by diastereoisomeric vicinal fluorohydrins (e.g., **52** and **53**, Figure 8) [94–95], marking this motif as a potentially valuable tool for molecular design.

Additional stabilisation of the *gauche* conformation of vicinal fluorohydrins can also be afforded by an intramolecular H-bond (**II**, Figure 8) [96–98]. Fluorine is a weaker H-bond acceptor than oxygen or nitrogen [99–101], but F···H attraction can still be significant in some contexts [102–103] and the intramolecular H-bond depicted in **II** (Figure 8) can have a significant impact upon the properties of vicinal fluorohydrins [97].

The possibility of intramolecular H-bonding in vicinal fluorohydrins has important consequences for the molecules’ intermolecular interactions too. The intramolecular H-bond makes the hydroxy group a weaker intermolecular H-bond donor [104] (e.g., **55** vs **54**, Figure 8). This runs counter to a longstanding assumption [105] that the inductive effect of fluorine should always make the hydroxy group a stronger H-bond donor. For non-rigid molecules, the greater the population of intramolecularly H-bonded conformers, the worse the hydroxy group will be as an intermolecular H-bond donor [106]. To be clear, fluorine can make the hydroxy group a stronger intermolecular H-bond donor in certain circumstances, for instance in highly-fluorinated alcohols (e.g., hexafluoroisopropanol) [107], or in rigid molecules where intramolecular H-bonding is not possible [104] (e.g., **56**, Figure 8); but it is not a universal phenomenon.

The issue of intra- vs intermolecular H-bonding of vicinal fluorohydrins can complicate matters if the hydroxy group is required for target binding. A series of (fluorinated) protease inhibitors illustrates this point (**57** and **58**, Figure 8) [108]. The non-fluorinated lead compound **57** is a natural product known as pepstatin; the backbone of **57** adopts a bent conformation when bound to the protease enzyme, and the hydroxy group of **57** interacts with catalytic aspartate residues in the active site. The fluorinated pepstatin analogue **58** was predicted to be pre-organised into the bent conformation and hence be a more potent inhibitor than **57**. Compound **58** was indeed found to be more potent than **57** (Figure 8), but the magnitude of the improvement was very small. This might be because fluorine delivered competing outcomes: on one hand, fluorine-derived conformational pre-organisation may have delivered an entropic benefit for target binding, but on the other hand, fluorine also may have caused a reduction of the H-bond-donor acidity of the hydroxy group of **58**. (See section 6, which focuses on carbonyl compounds, for a further explanation of the predicted conformation of **58**.)

Finally, two structural variations on the hydroxy group should be mentioned. First, if the hydroxy group is acylated (i.e., to generate an ester), the *gauche* O–C–C–F conformation is favoured over *anti* more strongly than was seen for the parent alcohol (e.g., energy difference between *gauche* and *anti* = 1.0 kcal·mol^−1^ for esters; 0.3 kcal·mol^−1^ for alcohols) [109]. This is likely due to enhanced hyperconjugation effects in the ester case (i.e., σ_C–H_ → σ*_C–F_ and σ_C–H_ → σ*_C–O_, **III**, Figure 8). Examples of this phenomenon are seen in the crystal structures of compounds **59** and **60** (Figure 8), which are synthetic precursors of β-fluorinated amphetamines; both of **59** and **60** feature a *gauche* O–C–C–F alignment regardless of other nearby functionality [110].

Second, if the hydroxy group becomes protonated under acidic conditions, the *gauche* ^+^O–C–C–F conformation becomes dramatically more favoured over *anti* (e.g., energy difference between *gauche* and *anti* = 7.2 kcal·mol^−1^). This is due to a new charge-dipole phenomenon (**IV**, Figure 8) that adds to the hyperconjugation and intramolecular H-bonding phenomena [96].

The conformational effects of fluorination in these two derivatives (i.e., esters and protonated alcohols) have been little exploited to date in the design of functional molecules [111]. However, one standout example that features both an ester moiety and an amino group will be discussed later, in section 5 of this review [112].

### 4 Sugars

We have examined several classes of molecules of gradually increasing complexity, progressing from alkanes (section 1) to ethers (section 2) and then alcohols (section 3). Throughout, we have seen that the introduction of fluorine can influence the molecular conformations in useful ways. We will now consider a more complex class of molecules in which the fluorine-derived conformational effects seen in all three of the preceding sections are united: namely, sugars.

Sugars are ubiquitous molecules in biology. Their functions permeate every aspect of life, including as essential components of oligonucleotide structure; as principal players in metabolism and energy storage; as motifs for the post-translational modification of proteins; and as partners in myriad supramolecular recognition processes. Therefore, methods for controlling the conformations of sugars are likely to have diverse and valuable applications in biotechnology and medicine.

The structure of a sugar molecule offers several potential locations where fluorine can be introduced. Typical patterns include the introduction of –CHF– or –CF_2_– as a replacement for the ring oxygen, or for one (or more) of the –CH(OH)– groups, or for the anomeric oxygen. The various conformational outcomes that flow from making such modifications are discussed below. It should be noted that introducing fluorine into sugars has many other effects, too, e.g., modulating lipophilicity [44,113–115], enabling target-binding interactions to be elucidated [116–117], or providing the opportunity for ^19^F and ^18^F imaging. Other reviews have covered many of these aspects [118–119], but here we will focus exclusively on the conformational aspects.

**Figure 9:**
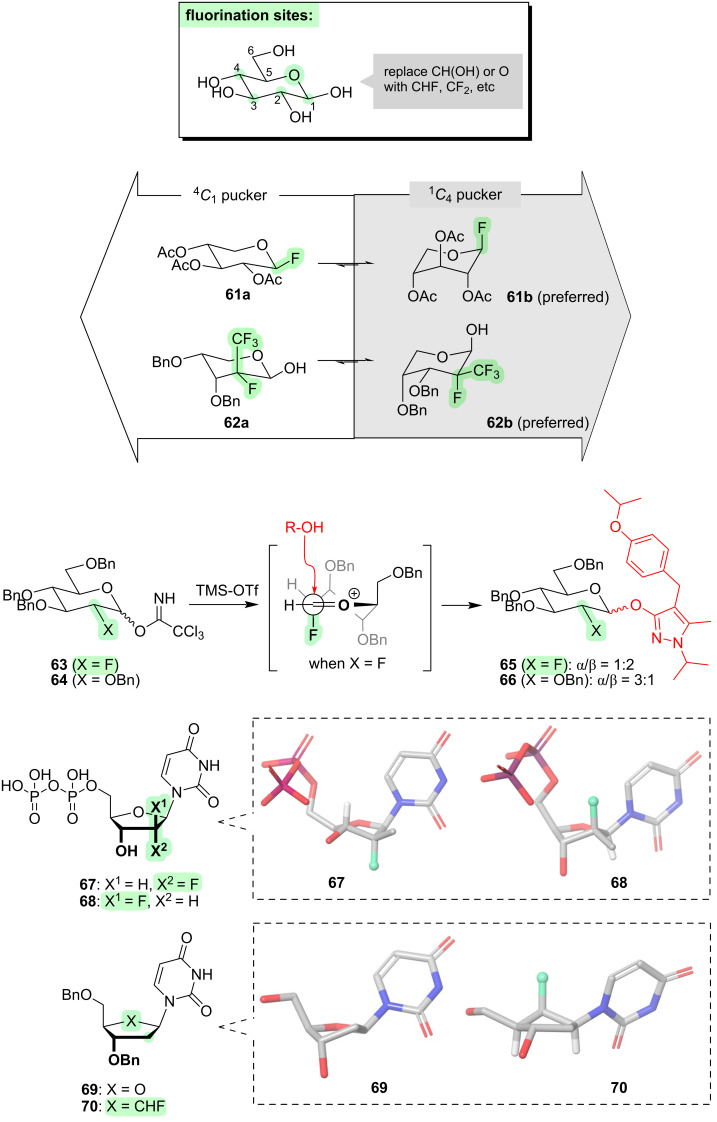
Controlling the ring pucker of sugars through fluorination.

We will first consider how fluorination can affect the ring pucker (Figure 9).

For pyranoses (i.e., six-membered ring sugars), the classic ^4^*C*_1_ chair conformation is usually strongly preferred. This conformation tends to be maintained even if one or several fluorines are introduced as replacements for hydroxy groups at positions C-1 and/or C-2 and/or C-3 and/or C-4, regardless of stereochemistry and even if 1,3-difluoro repulsion is incurred [44,117,120–122]. However, there are certain special circumstances where fluorination can invert the pucker. For example, in pentopyranoses (i.e., sugars lacking an exocyclic carbon), the ring pucker can be inverted by placing fluorine at C-1 with appropriate stereochemistry, due to a strong anomeric effect (**61**, Figure 9) [123]. In another example, the ring pucker of a pentopyranose was observed to be inverted when the –CH(OH)– moiety at C-2 was replaced with a –C(F)(CF_3_)– moiety with appropriate stereochemistry (i.e., **62**, Figure 9) [124]; in this case, the bulky CF_3_ group presumably has a strong tendency to occupy the equatorial position.

Up to now, we have seen that fluorine has a rather limited ability to influence the pucker of the six-membered ring of pyranoses. However, it must be remembered that pyranoses are reactive molecules that can undergo, e.g., glycosylation, and it emerges that fluorine can play a much more significant conformational role during the dynamics of such chemical reactions [125–130]. Glycosylation reactions (e.g., **63** → **65** and **64** → **66**, Figure 9) proceed via an oxocarbenium intermediate. If fluorine is located at C-2, an electrostatic attraction might be expected between the partially negative fluorine and the positively charged C=O^+^ moiety, and this interaction would favour one of the half-chair conformations of the oxocarbenium intermediate (Figure 9) [131–132]. Also, in the preferred pucker, the C–F bond is oriented orthogonal to the C=O group, which makes the carbonyl more electrophilic through mixing of the π*_C=O_ orbital with the σ*_C–F_ orbital. These combined effects enhance both the rate and the β-stereoselectivity of the glycosylation reaction (e.g., **63** → **65** vs **64** → **66**, Figure 9) [129]. Of course, the fluorine substituent remains in the product, which may or may not be advantageous depending on the particular biological context.

Let us now consider furanoses (i.e., five-membered ring sugars). Five-membered rings are inherently more flexible than six-membered rings, so there is more scope for fluorine to influence the pucker. When fluorine is introduced anywhere across C-1–C-3 as a replacement for a hydroxy group (e.g., **67** and **68**, Figure 9), the preferred ring pucker is dictated by the maximisation of σ_C–H_ → σ*_C–F_ hyperconjugative interactions, and this affords different puckers depending on the fluorine stereochemistry [133]. In this sense, the fluorine substituents are performing a similar role to a hydroxy group, so this is a “conformationally conservative” situation. It should be noted that one of the reasons why C-2 fluorinated nucleosides (e.g., **67** and **68**) have become especially popular in the field of medicinal chemistry [133–137], is that the presence of fluorine at C-2 confers enhanced stability towards hydrolysis, through destabilising the oxocarbenium intermediate [136].

Another way that fluorine can alter the shapes of furanoses, is by replacing the ring oxygen. For example, compare the nucleotide derivatives **69** and **70** (Figure 9). When the ring oxygen of **69** is replaced with a fluoromethylene group in **70**, the latter molecule adopts an unusual envelope conformation in which the fluorinated carbon is projected above the ring plane, stabilised by dual σ_C–H_ → σ*_C–F_ hyperconjugation [138].

**Figure 10:**
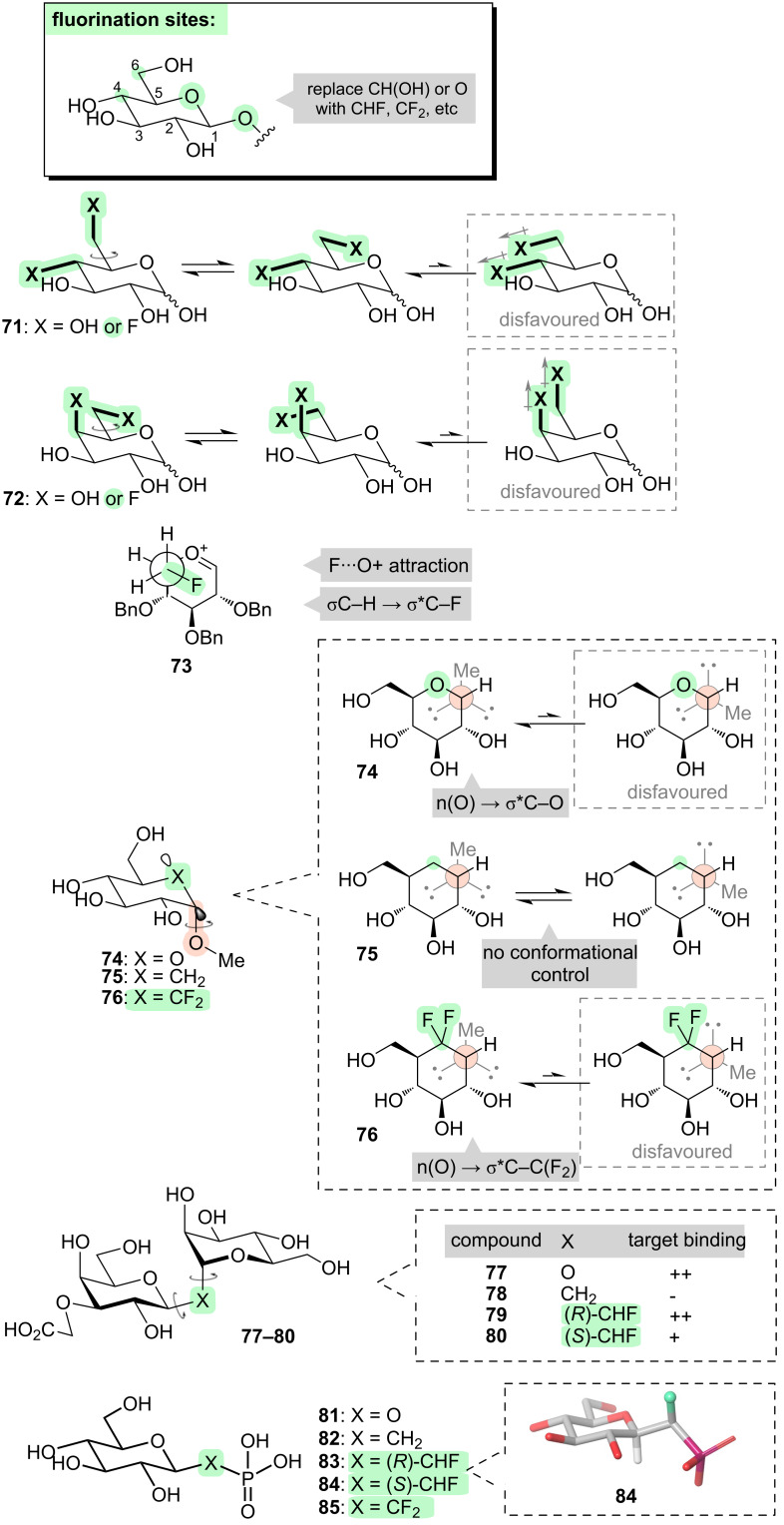
Controlling bond rotations outside the sugar ring through fluorination.

Let us now consider ways in which fluorine can influence bond rotations outside the sugar ring (Figure 10). We will focus initially on the C-5–C-6 bond. In natural sugars, rotation about the C-5–C-6 bond is influenced by the stereochemistry at C-4, because a parallel alignment of the 1,3-hydroxy groups at C-4 and C-6 is unfavourable. Fluorine can achieve the same effect whether located at C-4, or C-6, or both (e.g., **71** and **72**, Figure 10) [119]. Another way in which fluorine can control the rotation of the C-5–C-6 bond is seen when a C-6-fluorinated sugar is converted into an oxocarbenium ion (e.g., **73**, Figure 10). The C-6–F bond of **73** preferentially orients over the sugar ring, due to a combination of electrostatic attraction between the partially negative fluorine atom and the positively charged C=O^+^ moiety, and σ_C–H_ → σ*_C–F_ hyperconjugation. The fluorine atom thus shields the top face of the oxocarbenium ion, and this has flow-on effects on the rate and stereoselectivity of subsequent glycosylation reactions [139].

Another important rotatable bond that lies outside the sugar ring, is the C-1–O anomeric bond in glycosides (highlighted in **74**, Figure 10). This anomeric bond serves as a linker between the sugar and the rest of the glycoside; rotation about this bond can dramatically change the overall shape of the molecule, which in turn can affect its target-binding ability [140]. Fluorine can be used to control the rotation of the anomeric bond, through several means as described below.

“Carbasugars” are sugar analogues in which the ring oxygen is replaced with carbon. We have already seen an example of such a structure (i.e., **70**, Figure 9). Carbasugars are of broad interest because they can be used to create non-hydrolysable glycoside mimics; however, they are poor mimics of genuine glycosides in terms of the rotational profile about the anomeric bond. In true glycosides (e.g., **74**, Figure 10), the anomeric bond typically does not rotate freely; instead, it is biased towards one particular rotamer due to n_O_ → σ*_C1–O5_ hyperconjugation. This opportunity for hyperconjugation is lost with the carbasugar glycosides, and hence the anomeric bond in **75** tends to rotate more freely and this can compromise supramolecular binding interactions. However, the natural rotameric profile can be restored by replacing the –CH_2_– moiety of the carbasugar with a –CF_2_– moiety (e.g., **76**, Figure 10), because the electron-withdrawing character of the fluorine substituents enables reasonably effective n_O_ → σ*_C1–C(F)_ hyperconjugation to occur [141].

Another way to influence the rotameric profile of the anomeric bond in glycosides, is to replace the anomeric oxygen with a (fluorinated) carbon. For disaccharides (e.g., **77**, Figure 10), we have already seen that the lone pairs on the anomeric oxygen play a role in restricting rotation about the anomeric bond, due to n_O_ → σ*_C1–O5_ hyperconjugation. Therefore, if the anomeric oxygen is replaced with CH_2_ (e.g., **78**, Figure 10), the anomeric bonds are able to rotate more freely, and this manifests in a reduced binding affinity for the protein target by **78** compared to **77**. However, upon progressing to a CHF linkage (e.g., **79** and **80**, Figure 10), the molecule becomes more rigid again, this time due to dual σ_C–H_ → σ*_C–F_ hyperconjugation. Notably, different conformations are preferred by the epimeric fluorinated analogues **79** and **80**: analogue **79** is a good match for the parent disaccharide and retains its protein-binding affinity, whereas **80** loses some affinity [142].

A related class of compounds are the phosophosugars (e.g., **81**, Figure 10). Phosphosugars play important roles in a variety of metabolic processes, as well as constituting the backbone of oligonucleotides. Sugar phosphonates (i.e., with a CH_2_ linkage between sugar and phosphorus, e.g., **82**, Figure 10) are of interest as non-hydrolysable isosteres, but a better match in terms of p*K*_a_ and C–C–P angle is achieved with mono- or difluorophosphonates (e.g., **83**–**85**, Figure 10) [143–144]. In the case of the monofluorophosphonates, conformational effects can also be important [145–147]. For example, the diastereoisomeric monofluorophosphonates **83** and **84** were compared in their ability to bind to a phosphosugar-processing enzyme. Epimer **84** was found to bind with 100-fold higher affinity than epimer **83**, and this was attributed to a lower-energy binding conformation in the case of **84** which benefited from σ_C1–H_ → σ*_C–F_ hyperconjugation [146–147].

### 5 Amines

We now turn to a very important and diverse class of molecules: the amines. Nitrogen-containing compounds are highly represented in many fields, including the pharmaceutical and agrochemical industries. It transpires that incorporating fluorine atoms nearby to nitrogen can affect the molecular properties of amines in several useful ways, notably including their conformations. In this section we will commence by examining fluorine-derived conformational control in simple linear amines, then we will move on to cyclic amines. Next, we will examine some important derivatives of amines, such as amides and sulfonamides. Throughout, the emphasis will mostly be on bioactive molecules, but finally this section will conclude by examining a different type of molecular function, namely, organocatalysis.

**Figure 11:**
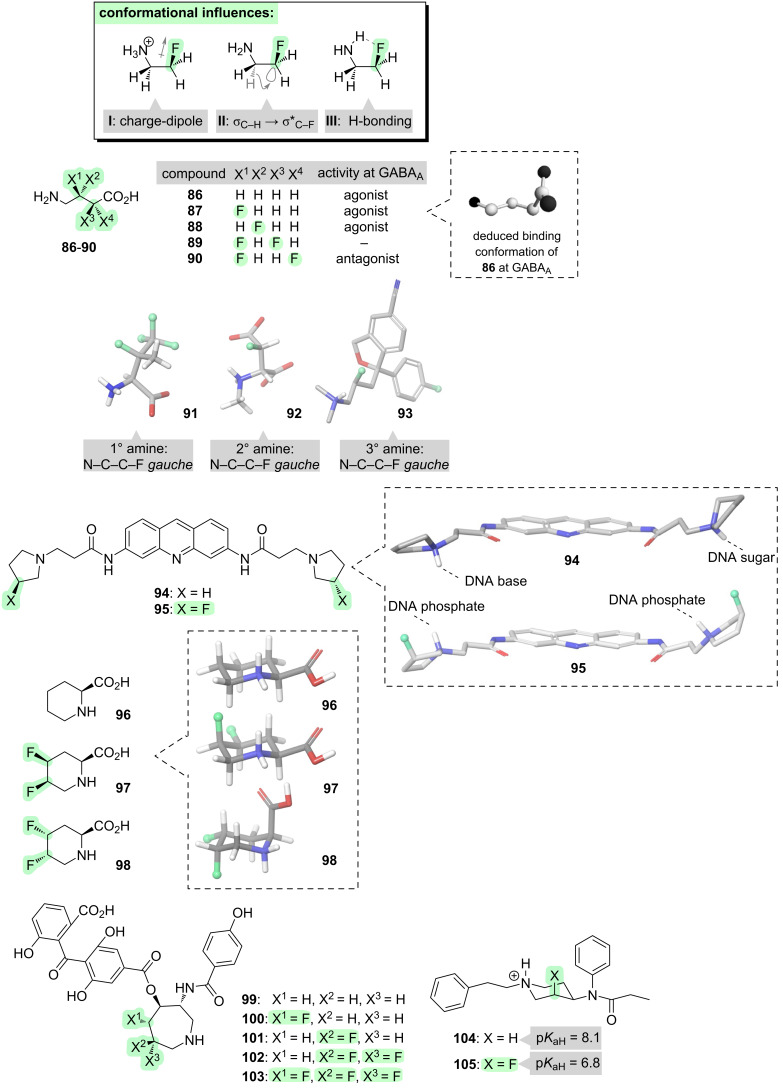
Effects of incorporating fluorine into amines.

When fluorine is positioned beta to nitrogen, the fundamental stereoelectronic interactions that arise (**I**–**III**, Figure 11) are conceptually similar to those that we have already seen with oxygen-containing molecules. The dominant interaction is an electrostatic attraction between the amine, which is typically protonated at neutral pH, and the partially negative fluorine atom (**I**, Figure 11) [96]. This attraction has the outcome of favouring a *gauche* F–C–C–N^+^ alignment. This *gauche* alignment can be further stabilised by two additional interactions: hyperconjugation (**II**, Figure 11) [148], and intramolecular hydrogen bonding (**III**, Figure 11) [149].

These effects have been exploited to control the conformations of simple bioactive amines such as γ-aminobutyric acid (GABA, **86**, Figure 11) [150–155]. GABA is a neurotransmitter that binds to a variety of different GABA receptors, and information about the target-binding conformations of GABA at these various receptors can be gleaned by investigating fluorinated GABA analogues (e.g., **87**–**90**). For example, the enantiomeric analogues **87** and **88** have similar activity at the GABA_A_ receptor, and this suggests that the binding conformation of GABA has an extended N^+^–C–C–C segment, since both fluorinated analogues **87** and **88** have a favourable *gauche* alignment of the C–N and C–F bonds in this conformation [150]. Further information can be obtained by incorporating a second fluorine atom. The difluorinated analogue **89** is found to have no activity at GABA_A_ whereas the difluorinated analogue **90** has some activity, and this suggests that the binding conformation of GABA has a bent C–C–C–C(O) segment since this conformation is disfavoured for **89** and favoured for **90** [151].

The β-fluoroamine *gauche* preference has been exploited to control the shapes, and hence to elucidate the bioactive conformations, of other simple linear amines too. A *gauche* alignment of the vicinal C–N and C–F bonds is consistently seen [156], whether the scaffold is a primary amine (e.g., tetrafluorovaline, **91** [157]), a secondary amine (e.g., *N*-methyl-ᴅ-aspartate(**92)** [158–159]), or a tertiary amine (e.g., a fluorinated analogue of the antidepressant drug, citalopram **93** [160]).

We now turn our attention to cyclic amines. Fluorine has been shown to be capable of modifing the conformations of a variety of *N*-heterocycles, ranging in size from four- to eight-membered rings [48,161–163]. In each case, the conformational outcome is determined by an interplay between electrostatic effects (**I**, Figure 11), hyperconjugation (**II**, Figure 11), intramolecular H-bonding (**III**, Figure 11), and sterics. For example, consider the G-quadruplex ligand **94** (Figure 11). This molecule contains two pyrrolidine moieties, the NH groups of which interact with the DNA bases and sugars, respectively. When fluorine is incorporated beta to each of the pyrrolidine nitrogens (i.e., **95**, Figure 11), the pucker of each ring changes due to electrostatic attraction between the partially negative fluorine and the protonated amine. These conformational changes alter the DNA-binding mode, such that the pyrrolidine NH groups of **95** now interact with the DNA phosphates rather than the bases or the sugars [164].

Progressing now to a six-membered ring system, consider the scaffold, pipecolic acid (**96**, Figure 11) [165–166]. This molecule is a ring-expanded analogue of proline, and derivatives of it are found within several natural products and drugs. The pucker of the six-membered ring of **96** is quite important for bioactivity, because different puckers project the carboxyl substituent in different orientations (equatorial vs axial). Normally, the pucker with an equatorial carboxyl group is favoured. This pucker is maintained in the difluorinated analogue **97** (Figure 11); in this conformation, in addition to the favourable equatorial placement of the carboxyl group, all of the interactions **I**–**III** (Figure 11) are satisfied. In contrast, the diastereoisomeric difluorinated analogue **98** favours the opposite pucker; in this case, the electrostatic attraction between the partially negative fluorine and the protonated amine (**I**, Figure 11) outweighs the equatorial preference of the carboxyl group.

Another cyclic amine in which the conformation can be controlled by fluorine, is the natural product balanol (**99**, Figure 11) [112,167–172]. Balanol is an ATP mimic that inhibits protein kinase Cε (PKCε), an enzyme that is implicated in cancer. However, compound **99** also inhibits off-target kinases including protein kinase A (PKA). Fluorination was investigated as a strategy for altering the conformation of the central seven-membered nitrogen heterocycle, and hence possibly improving the selectivity for PKCε. A variety of fluorination patterns were investigated (**100**–**103**, Figure 11), which were found to induce subtly different conformations of the seven-membered ring through an interplay of the conformational influences **I**–**III** (Figure 11) and sterics. Promisingly, these different shapes were found to successfully alter the PKCε/PKA selectivity, with one analogue (**100**) displaying higher potency and selectivity for PKCε than balanol itself [112]. This key result was alluded to earlier (in section 3 of this review, during the discussion of acylated alcohols), since compound **100** also features a F–C–C–O(acyl) moiety.

We conclude our examination of amines by considering a final impact of fluorination, which is that the amino group becomes less basic when an inductively electron-withdrawing fluorine atom is nearby. This effect can be exploited to adjust the charge-state of a drug molecule, which can help in optimising the drug’s target-binding and/or bioavailability [154,173–181]. For example, consider the analgesic drug fentanyl (**104**, Figure 11). This drug contains a piperidine moiety, which is protonated at physiological pH and forms a salt bridge with an aspartate residue in the binding site of the μ-opioid receptor. One of the drawbacks of fentanyl (**104**) is that it is not selective towards injured tissue: it also binds to μ-opioid receptors in healthy tissue, causing side-effects. To overcome this drawback, a fluorinated analogue of fentanyl ((±)-**105**) was developed [177]. The electron-withdrawing fluorine substituent of **105** lowers the p*K*_aH_ of the piperidine moiety to 6.8, such that it is only protonated at acidic pH. Since low pH is a hallmark of injured tissue, compound **105** maintains analgesic activity at the site of injury while offering fewer side-effects.

The magnitude of the p*K*_aH_-lowering effect varies with the number of fluorines, and with their proximity to the amino group. But there is another aspect to the modulation of p*K*_aH_ of amines by fluorine: conformation matters, too [182]. If the F–C–C–N alignment is *gauche* (as in **105**, Figure 11), the p*K*_aH_ will be lowered by ≈1 log unit compared to the non-fluorinated amine. However, if the F–C–C–N alignment is *anti*, the effect is stronger and the p*K*_aH_ will be lowered by ≈2 log units. Clearly it is important to bear this in mind when utilising fluorine to optimise the properties of nitrogen-containing drugs.

Having concluded our examination of fluorinated amines, let us now focus upon some important derivatives of amines, namely amides and sulfonamides.

**Figure 12:**
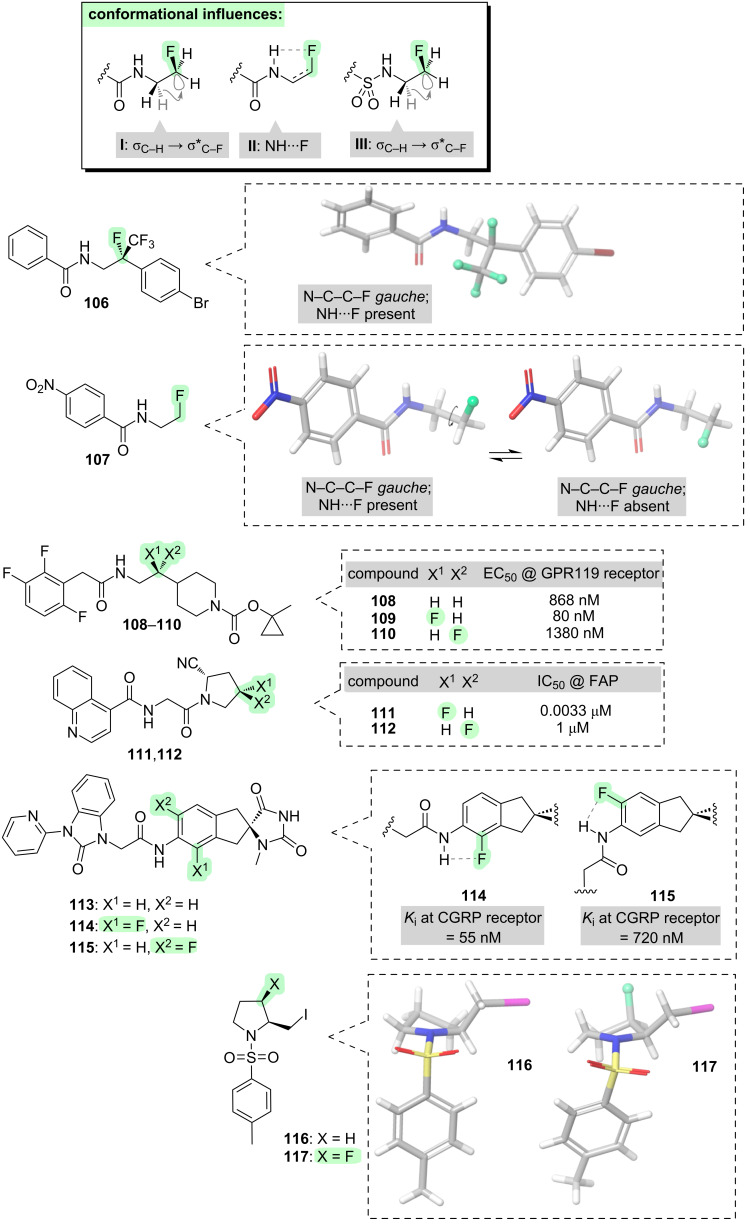
Effects of incorporating fluorine into amine derivatives, such as amides and sulfonamides.

Model studies with simple β-fluoroamides (e.g., **106** and **107**, Figure 12) reveal that such molecules consistently adopt conformations in which the F–C–C–N dihedral angle is *gauche* [183–185]. This situation is reminiscent of that already seen with β-fluoroamines, although the predominant driving force with β-fluoroamides is now hyperconjugation (**I**, Figure 12). For secondary amides, i.e., those that contain an NH group, the nitrogen-bound hydrogen sometimes makes a close contact with the fluorine (e.g., **106**), but this not always the case (e.g., **107**), suggesting that the NH···F interaction (i.e., **II**, Figure 12) is not a dominant one.

The *gauche* preference of β-fluoroamides has been exploited to control the conformations of a variety of bioactive molecules [186]. For example, the activity of the GPR119 receptor agonist **108** (Figure 12) can be modulated through fluorination [187]: either an increase or a decrease in potency is seen dependent on the fluorine stereochemistry, suggesting that the bioactive conformation is favoured for one stereoisomer (**109**) and disfavoured in the other (**110**). Another context in which the β-fluoroamide *gauche* effect has frequently been exploited is *N*-acylpyrrolidines, a structural motif found within many bioactive molecules (e.g., the fibroblast activation protein (FAP) inhibitors **111** and **112**, Figure 12) [188–192]. When fluorinated analogues of *N*-acylpyrrolidines are prepared, dramatic differences in biological activity are sometimes seen between the fluorinated stereoisomers (e.g., **111** and **112**) [189]. This difference can be attributed to a conformational effect, whereby fluorine controls the pucker of the 5-membered ring through σ_C–H_ → σ*_C–F_ hyperconjugation. Further examples of this phenomenon will be presented in section 7 (peptides).

It was stated above that the NH···F interaction (i.e., **II**, Figure 12) is not dominant. However, this interaction has been successfully exploited within the slightly different structural context of anilides (e.g., **113**–**115**, Figure 12) [193]. Anilide **113** is a GPCR receptor antagonist. The fluorinated analogues **114** and **115** were found to have very different levels of potency, attributed to different conformations due to the NH···F interaction. This information was subsequently applied to generate next-generation antagonists with even higher potency than **114** (not shown), by building a new ring structure onto the molecule to reinforce the optimal conformation suggested by **114**.

Related to amides are the sulfonamides (e.g., **III**, Figure 12). The sulfonamide functional group is found within a wide variety of important bioactive compounds. There is emerging evidence that the conformations of sulfonamides can be productively influenced through fluorination in a similar manner to that seen with amides. For example, the pucker of an *N*-tosylpyrrolidine **116** can be altered through fluorination (**117**), due to a preference of the F–C–C–N moiety to adopt a *gauche* conformation [194]. Suprisingly, this “fluorine-sulfonamide *gauche* effect” appears to have been exploited only very rarely in molecular design [194–198], suggesting that there may be considerable scope for this under-utilised conformational tool to be applied more widely in the future.

Throughout this section focusing on amines and their derivatives, most of the applications of fluorine-derived conformational control have been in the bioactives space. To conclude this section, a different application will be examined, namely organocatalysis.

**Figure 13:**
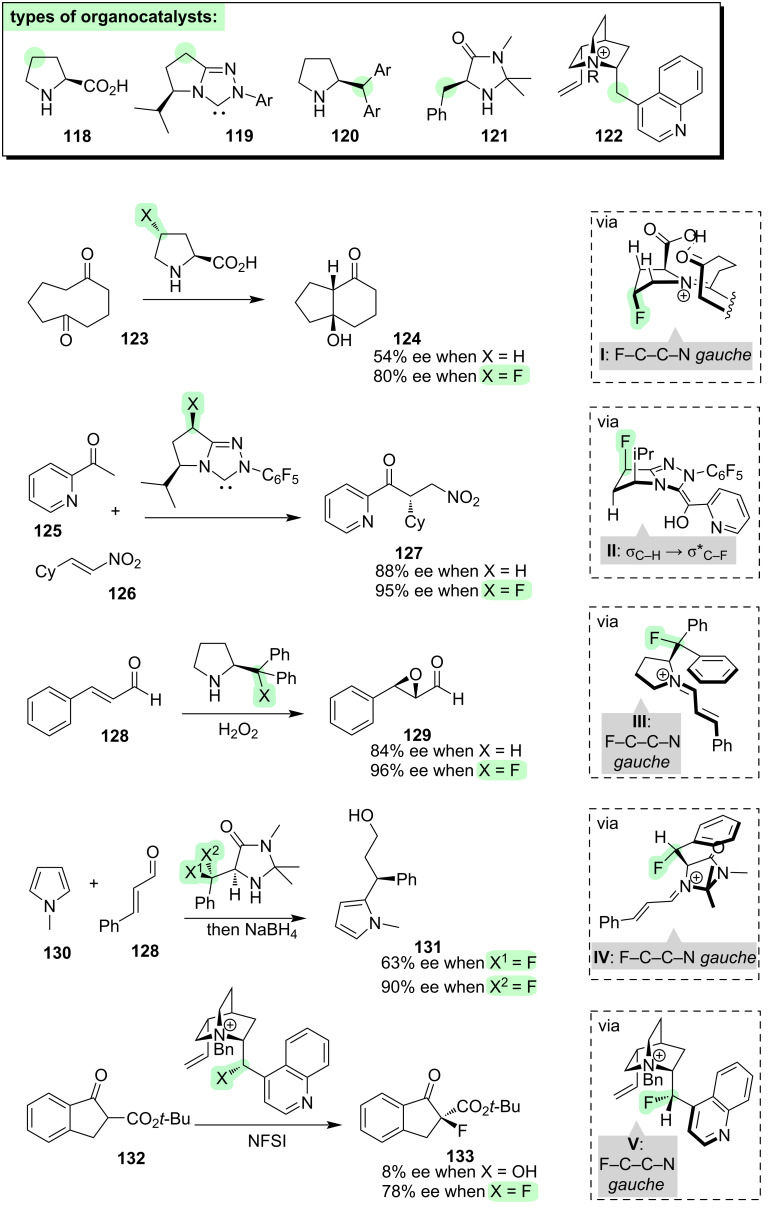
Effects of incorporating fluorine into organocatalysts.

Some important examples of organocatalysts are shown in Figure 13, including proline (**118**), an *N*-heterocyclic carbene (NHC, **119**), a proline derivative **120**, an imidazolidinone **121**, and a cinchona alkaloid **122**. These structures are quite diverse, and they operate via different catalytic mechanisms, but they are all *N*-heterocycles and they all offer the opportunity for conformational modulation through fluorination (see green highlights in **118**–**122**) [199–200].

The first general strategy is to employ fluorine to control the ring pucker of the organocatalyst [36,201–205]. For example, incorporating fluorine at the 4-position of proline increases the enantioselectivity of a proline-catalysed transannular aldol reaction (**123 → 124**, Figure 13) [206]. The improved selectivity of the fluorinated catalyst compared to proline itself can be attributed to conformational organisation of the activated intermediate (**I**, Figure 13), due to σ_C–H_ → σ*_C–F_ hyperconjugation and electrostatic attraction between the partially negative fluorine and the C=N^+^ moiety. In another example, incorporation of fluorine into a NHC catalyst enhances the enantioselectivity of an intermolecular Stetter reaction (**125 → 127**, Figure 13). The improved selectivity can again be attributed to σ_C–H_ → σ*_C–F_ hyperconjugation, which imposes a curvature onto the Breslow intermediate (**II**, Figure 13).

The second general strategy is to employ fluorine to control rotation of the organocatalyst’s exocyclic bonds [202,207–216]. For example, a fluorinated proline-derived catalyst delivers high enantioselectivity in an epoxidation reaction (**128 → 129**, Figure 13). The high selectivity can be attributed to rigidification of the activated intermediate through the “fluorine–iminium *gauche* effect” (**III**, Figure 13), with one of the aryl groups effectively shielding the front face of the molecule [208]. A similar concept is seen with imidazolidone-type catalysts in the context of a Michael reaction (**130 → 131**, Figure 13) [214]. Fluorine can be used to bias the rotation about an exocyclic C–C bond of the catalyst, due to electrostatic attraction between the partially negative fluorine and the C=N^+^ moiety, and comparing the performance of the fluorinated catalysts then provides information about which rotamer is optimal for enantioselectivity (**IV**, Figure 13). A final example of the control of exocyclic bond rotations of an organocatalyst is seen with the cinchona alkaloids, operating as phase-transfer agents in the fluorination reaction of a β-ketoester (**132 → 133**, Figure 13) [212]. The presence of fluorine within the catalyst restricts the rotation of an exocyclic bond, due to electrostatic attraction between the partially negative fluorine and the C=N^+^ moiety (**V**, Figure 13), and this delivers superior enantiocontrol compared to the non-fluorinated catalyst.

### 6 Carbonyl compounds

**Figure 14:**
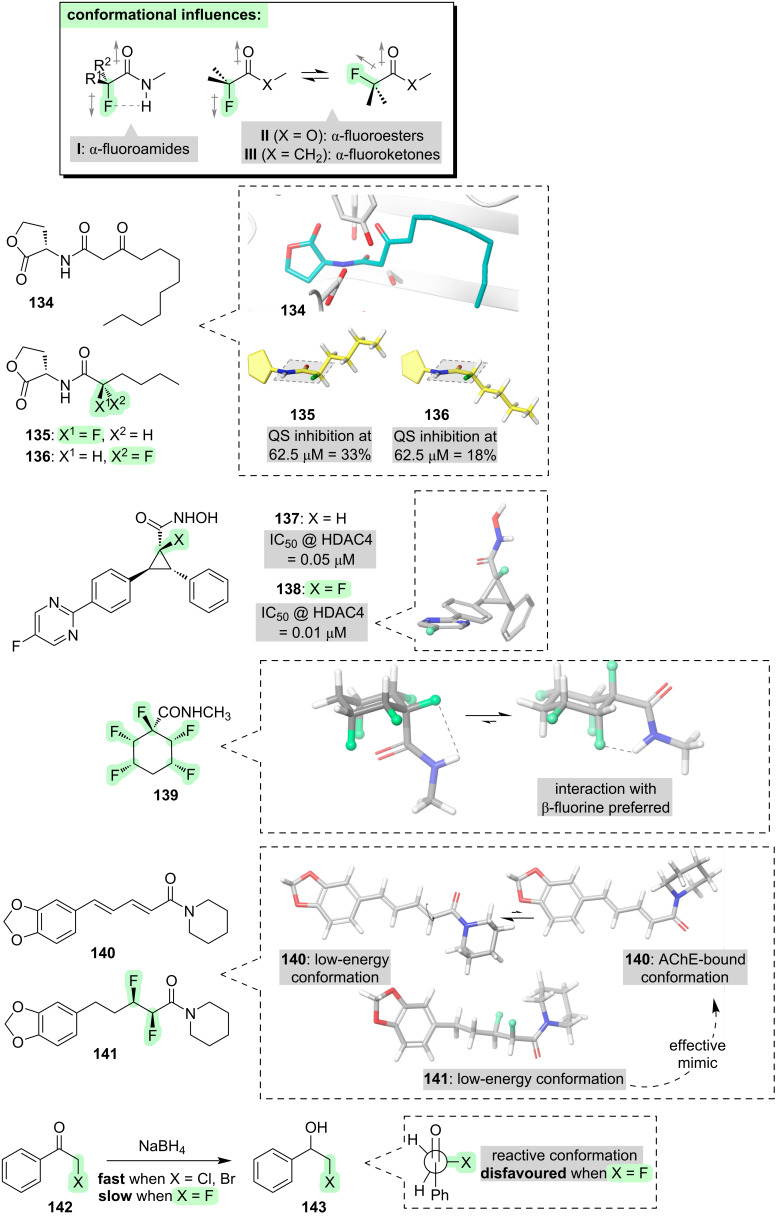
Effects of incorporating fluorine into carbonyl compounds, focusing on the “carbon side.”

We will now turn our attention towards molecules that contain a C=O double bond. We previously saw examples of such molecules when we were considering acylated derivatives of alcohols (section 3) and amines (section 5); however, in those cases we were considering the effect of fluorination on the “oxygen side” or the “nitrogen side” of the carbonyl group, whereas in this section we will consider the effect of fluorination on the “carbon side” (Figure 14).

If a fluorine atom is positioned at the α-position of a secondary amide (i.e., **I**, Figure 14), there is a strong tendency for the C–F and C=O bonds to align antiparallel. This tendency can be rationalised through a combination of dipole minimisation, and attraction between the fluorine and the nitrogen-bound hydrogen [217–220]. Notably, the conformation depicted in **I** (Figure 14) causes the carbon chain of the amide to deviate from the usual zigzag shape: the substituents R^1^ and R^2^ are projected in front of, and behind, the amide plane, respectively. This phenomenon has been exploited to control the conformations of bioactive molecules. For example, amide **134** (Figure 14) is an autoinducer involved in the bacterial quorum sensing (QS) response. The fluorinated analogues **135** and **136** were designed as QS inhibitors [221]. In analogue **135**, the fluorine substituent causes the carbon chain to be projected above the amide plane, successfully mimicking the receptor-bound conformation of the natural ligand **134**, and it emerges that analogue **135** is a more potent QS inhibitor than the stereoisomeric analogue **136**. Another illustration of this conformational phenomenon is seen with the HDAC inhibitors **137** and **138** (Figure 14) [222]. The fluorinated analogue **138** is more potent, and this can be attributed to fluorine-derived conformational preorganisation of the key hydroxamic acid moiety in **138**.

Further examples of the “α-fluoroamide effect” will be seen in section 7 (peptides).

It was stated above that the preferred conformation of an α-fluoroamide can be attributed to two separate phenomena, i.e., dipolar minimisation and F···HN attraction (**I**, Figure 14). It is possible to dissect these two phenomena through the study of structural variations. For example, in compound **139** (Figure 14) there are additional fluorine substituents one carbon further away from the carbonyl group [223]. The lowest-energy conformation of **139** features a close contact between one of the β-fluorines and the NH group, speaking to the generality of the F···HN interaction. On the other hand, consider tertiary amides (e.g., **140** and **141**, Figure 14), in which there is no NH group. α-Fluorinated tertiary amides typically do not have a single, dominant conformation: instead, a variety of rotamers about F–C–C=O are accessible, governed by an interplay between dipolar effects and the avoidance of steric repulsion [224–228]. Compound **140** is the natural acetylcholinesterase (AChE) inhibitor, piperine [229]. At first glance, the fluorinated analogue **141** is a poor mimic of the low-energy conformation of **140**. Intriguingly, however, compound **141** retains or even exceeds the AChE inhibitory activity of **140**. This can be explained by considering that piperine (**140**) adopts a higher-energy conformation when bound to AChE [230], and this higher-energy conformation is successfully mimicked by **141**.

We now turn our attention towards other types of carbonyl compounds, namely esters and ketones (e.g., **II** and **III**, Figure 14). As was seen above for α-fluorinated tertiary amides, in α-fluorinated esters and ketones there is no possibility for any F···HN interaction. As might therefore be expected, no single dominant conformation of **II** and **III** is typically observed; instead, rotation about the F–C–C=O dihedral leads to twin minima at ≈0° and ≈180° [231–233]. These two conformations usually have quite similar energies but different dipole moments, and hence the polarity of the surrounding medium can influence which conformation predominates. The lack of a strong inherent conformational preference of α-fluorinated esters and ketones perhaps explains why biological applications of such molecules are quite rare [234]. There is, however, a notable effect in terms of chemical reactivity. α-Halogenated ketones are generally regarded as being good substrates for nucleophilic addition and substitution reactions (e.g., **142 → 143**, Figure 14), but perhaps surprisingly, α-fluorinated ketones are an exception and they display low reactivity. This can be explained by considering that the most reactive conformation of α-haloketones features an orthogonal alignment of the C–X and C=O bonds (Figure 14), in which mixing of the σ*_C–X_ and π*_C=O_ orbitals can occur; this conformation is disfavoured for α-fluoroketones due to a clash between the fluorine lone pairs and the π-orbital [233].

### 7 Peptides

In previous sections of this review, we examined the conformational effects of fluorination upon amides, either on the “nitrogen side” (section 5) or the “carbon side” (section 6). Now we will go further and examine some more complex oligoamide scaffolds, namely, peptides and proteins. These are archetypal systems in which the 3D structure determines function; hence, methods for controlling the conformations of peptides and proteins are likely to have valuable applications in biotechnology and medicine.

**Figure 15:**
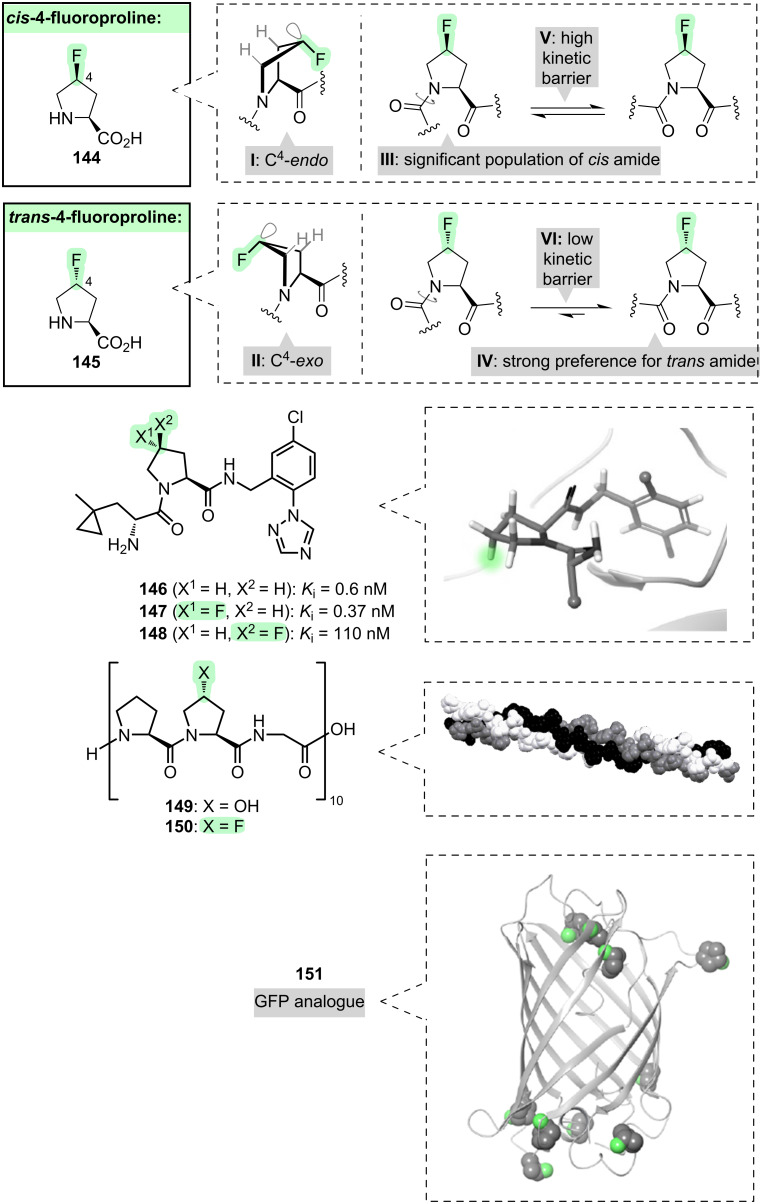
Fluoroproline-containing peptides and proteins.

The amino acid upon which most attention has been focused by the fluorine chemistry community to date, is proline (Figure 15).

Fluorination at the 4-position of the proline ring (**144** and **145**, Figure 15) has several conformational outcomes [235–239]. First, the pucker of the ring system can be biased towards either the C^4^-*endo* or the C^4^-*exo* conformation, depending on the fluorine stereochemistry (**I** and **II**, Figure 15): this can be attributed to σ_C–H_ → σ*_C–F_ hyperconjugation in each case. Second, the *cis*/*trans* ratio of the amide bond preceding the fluoroproline residue is affected: while *cis*-4-fluoroproline can accommodate a *cis*-amide configuration relatively easily (**III**, Figure 15), *trans*-4-fluoroproline strongly favours the *trans*-amide configuration due to n_O_ → π*_C=O_ hyperconjugation (**IV**, Figure 15). Third, the barrier to *cis*/*trans* isomerisation of the preceding amide bond is affected: *cis*-4-fluoroproline has a higher barrier to rotation while *trans*-4-fluoroproline has a lower barrier (**V** and **VI**, Figure 15), and this is consistent with the transition state of the isomerisation reaction having a C^4^-*exo* ring pucker.

These various conformational effects have been widely exploited to modulate the properties of proline-containing peptides and proteins [238–240].

The first example that we will consider involves a small peptide, and we will focus on the ring pucker. Peptide **146** is a thrombin inhibitor (Figure 15); its target-binding conformation features a C^4^-*exo* pucker of the proline ring [241]. Analogue **147**, which contains a *trans*-4-fluoroproline residue, is more potent than **146** because the proline ring of **147** is pre-organised into the required C^4^-*exo* pucker. By contrast, analogue **148**, which contains a *cis*-4-fluoroproline residue, is less potent.

The second example involves a larger peptide, and we will consider not just the ring pucker, but the thermodynamics and kinetics of *cis*/*trans* amide isomerisation, too. Peptide **149** (Figure 15) is a simplified and truncated model of the protein collagen. Like collagen, peptide **149** self-assembles into a triple helix in which all of the amide bonds are *trans* and all of the hydroxyproline residues have the C^4^-*exo* pucker. If the hydroxyproline residues of **149** are replaced with *trans*-4-fluoroproline (**150**, Figure 15), the resulting triple helix forms more quickly and is more stable [242]. The accelerated self-assembly of **150** can be attributed to a lowering of the *cis*/*trans* amide isomerisation barrier by fluorine (**VI**, Figure 15), while the greater thermodynamic stability of the self-assembled structure of **150** can be attributed, in part, to a stabilisation of both the *trans*-amide (**IV**, Figure 15) and the C^4^-*exo* pucker (**II**, Figure 15) by fluorine.

The third example illustrates how fluoroprolines can be used as tools for interrogating the structures and functions of entire proteins [243–254]. Green fluorescent protein (GFP, Figure 15) folds into a barrel shape, with the majority of its proline residues (nine out of ten) adopting the C^4^-*endo* pucker. When all of these prolines are replaced with *cis*-4-fluoroproline (**151**, Figure 15), the resulting protein maintains fluorescence properties and is a “superfolder”: it exhibits faster folding kinetics, and its folded state has enhanced stability [255]. This is consistent with the C^4^-*endo* pucker being favoured by *cis*-4-fluoroproline. In contrast, when all of the proline residues are replaced with *trans*-4-fluoroproline (not shown in Figure 15), the resulting protein fails to fold correctly and lacks fluorescence.

It is also possible to fluorinate at other positions of the proline ring. For example, in 3-fluoroprolines (not shown in Figure 15), analogous conformational effects are observed to those already described for 4-fluoroprolines, as would be expected since both series of molecules contain an N–C–C–F motif [256–257].

Let us now turn our attention away from fluoroprolines, and towards other types of fluorinated peptides.

**Figure 16:**
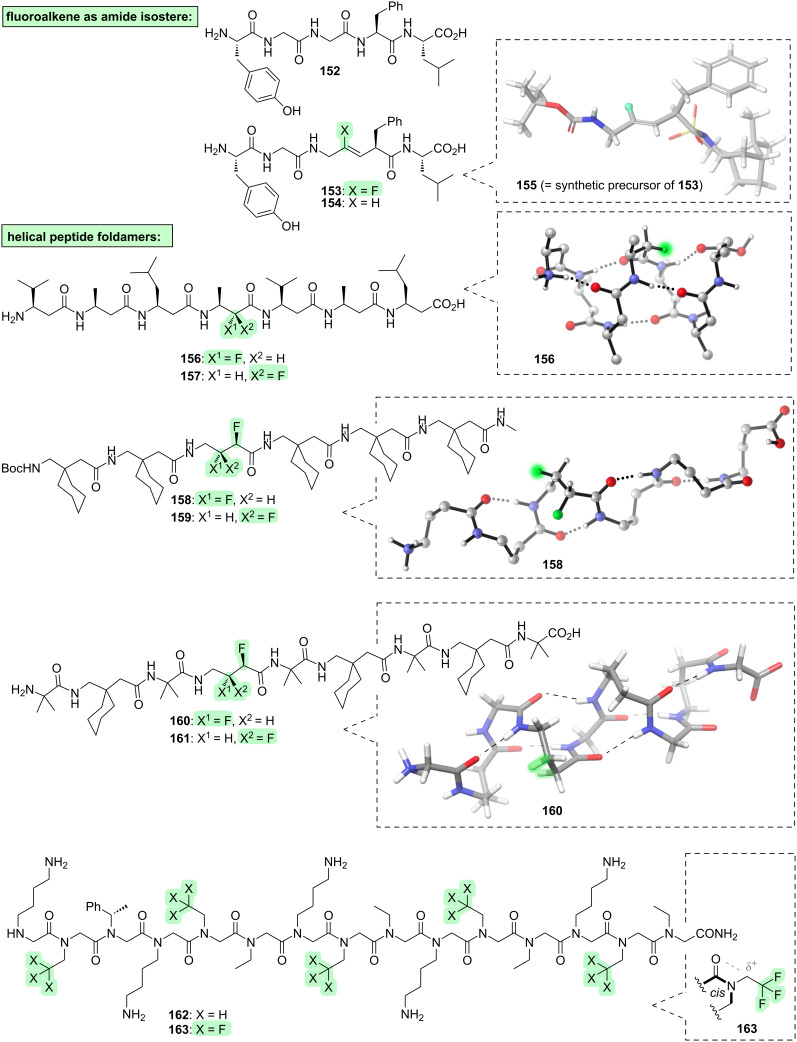
Further examples of fluorinated linear peptides (besides fluoroprolines). For clarity, sidechains are omitted from the 3D structures of **156**/**158**/**160**.

The fluoroalkene motif has frequently been employed within peptidomimetic structures, where it can serve as an effective isostere of the amide bond [258–261]. For example, a peptide known as Leu-enkephalin (**152**, Figure 16) is a potent ligand of the delta opioid receptor, but it has a poor pharmacokinetic profile due, in part, to its low lipophilicity. Replacement of one of the amide bonds of **152** with a fluoroalkene **153** increases the lipophilicity while retaining much of the receptor-binding activity [262]. In contrast, the non-fluorinated alkene analogue **154** loses receptor-binding activity, and this suggests that the fluorine in **153** serves the important function of mimicking the H-bond-accepting character of the carbonyl group of **152**. The fluoroalkene moiety of **153** is rigid, of course, so at first glance this example might appear to be outside the scope of this review, focusing as we are upon controlling molecular conformation. However, the fluoroalkene moiety of **153** does affect the conformation of a flanking segment of the peptidomimetic: in the synthetic precursor **155** (Figure 16), X-ray crystallography reveals a *gauche* alignment of the F–C–C–N moiety, as would be expected due to hyperconjugative stabilisation (σ_C–H_ → σ*_C–F_).

Another way to install fluorine on the backbone of a peptide, is to do so between the amino and carboxyl groups of one of the amino acids. This is difficult in the case of α-amino acids, because geminal fluoroamines are typically unstable [263]; but richer opportunities arise with backbone-extended amino acids such as β-, γ-, and δ-amino acids [264–270]. We have already seen an example of a backbone-fluorinated γ-amino acid (i.e., **58**, Figure 8, section 3); previously, compound **58** was considered as an example of a vicinal fluoro-alcohol, but now we can view the structure more holistically as a peptide that contains a backbone-fluorinated γ-amino acid, in which conformational control is exerted not just within the HO–C–C–F segment as discussed in section 3, but also within the F–C–C=O segment.

Another application of fluorinated backbone-extended amino acids is in the conformational control of helical peptide foldamers (Figure 16). For example, consider the β-peptides **156** and **157** [271]. These peptides differ only in the configuration of a single fluorinated stereocentre, yet they have dramatically different conformations: β-peptide **156** adopts a 14-helical shape, stabilised by favourable N–C–C–F and F–C–C=O alignments, whereas β-peptide **157** is forced out of the helical shape (not shown). Next, consider the γ-peptides **158** and **159** [272]. The *erythro*-difluorinated γ-peptide **158** adopts a 9-helical shape (Figure 16), due to favourable N–C–C–F and F–C–C–F and F–C–C=O alignments, whereas the *threo*-difluorinated γ-peptide **159** is disrupted away from this helical shape (not shown). A similar contrast between *erythro*- and *threo*-difluorinated stereoisomers is seen within the α,γ-hybrid peptide scaffold **160** and **161** [273]. Notably, the fluorines are the sole source of chirality in peptides **158**–**161**. Finally, consider the peptoids **162** and **163** [274]. The non-fluorinated peptoid **162** has the ability to adopt a helical conformation in which all of the amides are *cis*; but this helix is only a minor fraction of the total population of **162** in solution. The helical propensity is increased in the fluorinated analogue **163**, because the *cis*-amides are stabilised by a dipolar interaction between the carbonyl oxygen and the δ^+^ first carbon of the sidechain.

To conclude our survey of fluorinated peptides, we will focus upon cyclic examples.

**Figure 17:**
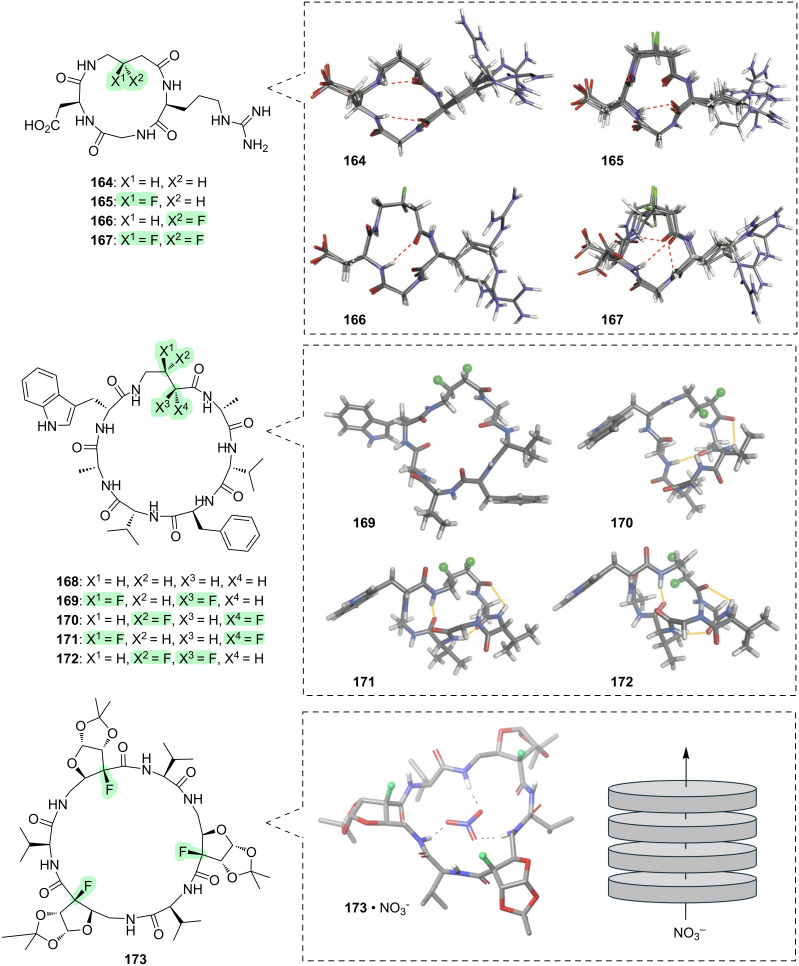
Fluorinated cyclic peptides.

An attractive feature of the cyclic peptide architecture, is that it becomes possible to control the shape of a bioactive epitope by altering a distant part of the macrocycle [275]. This concept has been explored with the cyclic RGD-containing peptides **164**–**167** (Figure 17) [276]. In these molecules, the shape of the integrin-binding RGD motif can be varied by changing the fluorination pattern within the γ-amino acid at the opposite side of the macrocycle. This alteration in the shape of the RGD motif translates into significant differences in the effects that **164**–**167** have upon cell adhesion and cell spreading. For example, cyclic peptides **164** and **165** inhibit the spreading ability of cells on a fibronectin-enriched extracellular matrix, while peptides **166** and **167** are less active; this suggests that a bent-shaped γ-turn about the glycine residue, as seen in **164** and **165**, is optimal for binding to fibronectin-recognising integrins such as α_V_β_3_ integrin.

Another cyclic peptide scaffold in which conformation has been altered by fluorination, is the natural product unguisin A (**168**, Figure 17). Incorporation of different 1,2-difluoro patterns within the γ-amino acid residue leads to different overall molecular shapes **169**–**172** [277–278], and these shape changes affect the ability of the cyclic peptide scaffold to act as a supramolecular host for binding a chloride anion guest [279].

The anion binding concept is taken further with the macrocycle **173** (Figure 17) [280]. This is a rather exotic cyclic hexapeptide comprising an alternating sequence of α- and γ-amino acids, and in which the γ-amino acids contain an embedded fluorosugar moiety. Peptide **173** adopts a “bracelet”-type conformation in which the macrocycle is quite flat and several of the amide bonds are oriented perpendicular to it, stabilised by an antiparallel alignment of each F–C–C=O moiety. This conformation enables supramolecular aggregation to form nanopores that are capable of transporting several different types of anions (e.g., NO_3_^−^, Cl^−^, SCN^−^, Br^−^) across a membrane.

### 8 Sulfur-containing compounds

For this final section, we will consider a class of compounds that is only just beginning to be explored in the context of fluorine-derived conformational control. Sulfur-containing compounds are found in a variety of important arenas, including as bioactives, as metal ligands, and as light-harvesting materials. Evidence is beginning to emerge that the conformations of sulfur-containing compounds can be controlled in valuable ways through fluorination.

**Figure 18:**
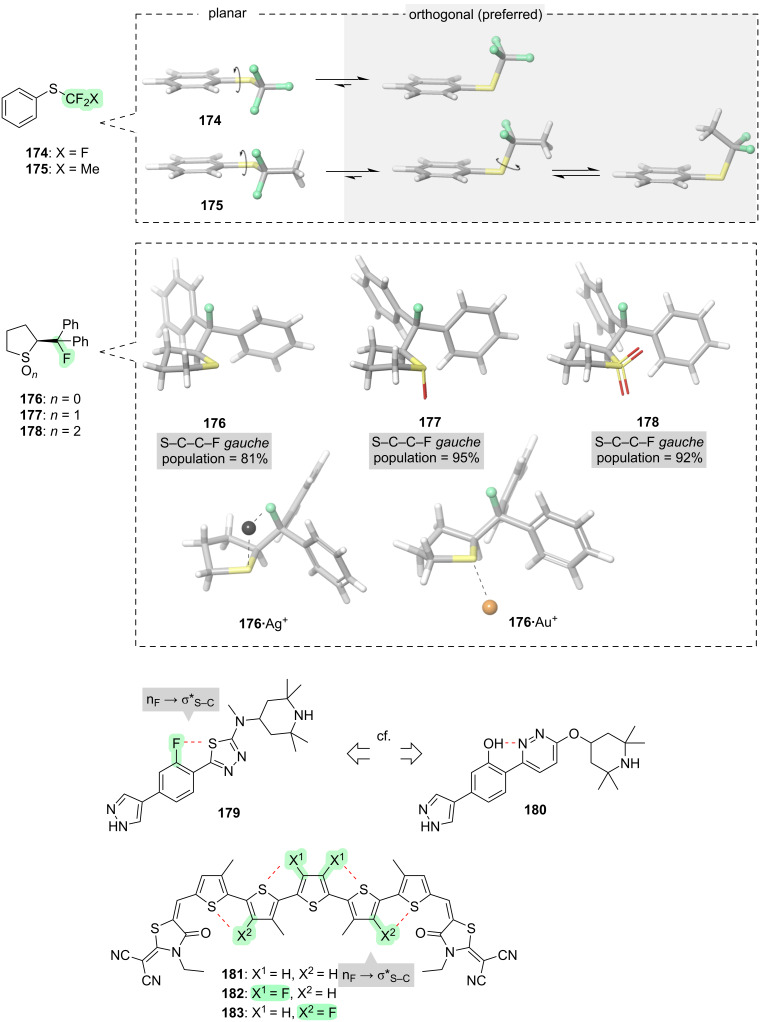
Fluorine-derived conformational control in sulfur-containing compounds.

In some instances, the conformational effects are similar to those that we have already seen elsewhere in this review. For example, consider the aryl thioethers **174** and **175** (Figure 18). By analogy with the oxygen ethers presented in section 2, fluorinated thioethers such as **174** and **175** tend to favour conformations in which the (Ar)C–S bond is orthogonal to the aryl system [281–282]. In the thioether case, this tendency can be attributed primarily to a steric effect rather than the hyperconjugation phenomenon that was discussed in the context of oxygen ethers.

Another fluorinated sulfur-containing compound in which the conformational properties should be somewhat familiar in light of previous discussions in this review, is the vicinal fluorothioether **176** (Figure 18). In compound **176**, there is a preference for the vicinal C–F and C–S bonds to align *gauche*, and this can be attributed to σ_C–H_ → σ*_C–F_ hyperconjugation [283]. Now, however, a new point of interest arises: the partial charge on sulfur can be manipulated in several different ways, and this can affect the conformation. For example, oxidation of **176** furnishes sulfoxide **177** or sulfone **178**, and in these oxidised analogues the “fluorine–sulfur *gauche* effect” is strengthened [283–284]. The stronger *gauche* effect in **177** and **178** is mostly attributable to a greater electrostatic attraction between fluorine and sulfur. Another way in which the partial charge on sulfur can be increased, and the *gauche* effect thereby strengthened, is through metal complexation (e.g., **176**·Ag^+^ and **176**·Au^+^, Figure 18) [285].

Another unique feature of sulfur-containing compounds is their ability to engage in attractive 1,5-F···S interactions. For example, compound **179** favours a planar conformation in which a lone pair on fluorine can mix with the σ* orbital of the S–C bond (Figure 18) [286]. This new type of hyperconjugation [287] enables compound **179** to successfully mimic the shape of a previously developed splicing modulator drug **180** while offering superior potency and pharmacokinetic properties. The 1,5-F···S interaction has also been exploited to control the conformations of oligothiophenes (e.g., **181**–**183**) [288], an important class of materials that have light-harvesting and semiconductor applications [289].

## Conclusion

We have seen that the C–F bond tends to align in predictable ways with neighbouring functional groups such as ethers, alcohols, amines, amine derivatives, carbonyl groups, and sulfur-containing moieties. In all cases, the favoured bond alignments can be understood in terms of simple stereoelectronic phenomena such as hyperconjugation and electrostatic attraction/repulsion.

These conformational effects can deliver several different types of advantages in functional molecules. In some instances, the C–F bond endows a molecule with the ability to change its polarity to suit its environment (a “conformational chameleon”). In other instances, the C–F bond allows a molecule to conservatively mimic the shape of a different molecule such as a natural product, while simultaneously offering some other kind of benefit (e.g., enhanced pharmacokinetic properties). In other instances, the C–F bond causes an otherwise flexible molecule to be rigidified, such that out of an ensemble of previously accessible conformations, a single conformation becomes dominant (application to entropy-driven enhancement of target-binding). Finally, in some instances the introduction of a C–F bond can cause a molecule to adopt an entirely novel shape.

Throughout this review, we have seen how such advantages have been expressed across a wide variety of molecular scaffolds, encompassing bioactive agents, functional materials, organocatalysts, peptide foldamers, supramolecular hosts, and even fragrance chemicals.

In the future, it seems likely that creative new expressions of these ideas will continue to arise, fuelled by ever-evolving synthetic fluorination methods [290]. It is the authors’ hope that our review article might contribute in a small way to this expansion, by inspiring readers to consider applying the principles of fluorine-derived conformational control to their own molecular scaffolds of interest.

## Data Availability

Data sharing is not applicable as no new data was generated or analyzed in this study.
